# Integrating network pharmacology, quantitative transcriptomic analysis, and experimental validation revealed the mechanism of cordycepin in the treatment of obesity 

**DOI:** 10.3389/fphar.2025.1571480

**Published:** 2025-05-14

**Authors:** Yu Liao, Mingchao Wang, Fuli Qin, Taotao Liu, Jiemei Chen

**Affiliations:** ^1^ Department of Pharmacy, The First Affiliated Hospital of Guangxi Medical University, Nanning, China; ^2^ Institute of Materia Medica, Chinese Academy of Medical Sciences and Peking Union Medical College, Beijing, China

**Keywords:** cordycepin, obesity, network pharmacology, quantitative transcriptomics, molecular docking, experimental validation

## Abstract

**Introduction:**

Evidence of the benefits of cordycepin (Cpn) for treating obesity is accumulating, but detailed knowledge of its therapeutic targets and mechanisms remains limited. This study aimed to systematically identify Cpn’s therapeutic targets and pathways in Western diet (WD)-induced obesity using integrated network pharmacology, transcriptomics, and experimental validation.

**Methods:**

A Western diet (WD)-induced mice model was used to evaluate the effectiveness of Cpn in ameliorating obesity. A network pharmacology analysis was then employed to identify the potential anti-obesity targets of Cpn. GO functional enrichment and KEGG pathway analysis were performed to elucidate the potential functions of the identified targets, followed by constructing a protein-protein interaction network to screen the core targets. Meanwhile, quantitative transcriptomics was conducted to validate and broaden the network pharmacology findings. Finally, molecular docking and quantitative real-time PCR assay were used for the core target validation.

**Results:**

Cpn treatment effectively alleviated obesity-related symptoms in WD-induced mice. The metabolic pathway, insulin signaling pathway, HIF-1 signaling pathway, FoxO signaling pathway, lipid and atherosclerosis pathway, and core targets including CPS1, HRAS, MAPK14, PAH, ALDOB, AKT1, GSK3B, HSP90AA1, BHMT2, EGFR, CASP3, MAT1A, APOM, APOA2, APOC3, and APOA1 are involved in regulating the therapeutic effect of Cpn.

**Conclusion:**

This study comprehensively uncovers the potential mechanism of Cpn against obesity based on network pharmacology and quantitative transcriptomics, which provides evidence for revealing the pathogenesis of obesity, suggesting that Cpn is a possible lead compound for anti-obesity treatment.

## 1 Background

Obesity is currently a major global health challenge, with its prevalence notably exacerbated in recent years. It is a chronic metabolic, inflammatory disease characterized by disrupted energy metabolism and abnormal fat accumulation. The well-established understanding now is that obesity notably increases the risks and disease burdens of insulin resistance, non-alcoholic fatty liver disease (NAFLD), type 2 diabetes mellitus, cardio-cerebral vascular disease, and even certain tumors (De et al., 2023). However, most drugs used to treat obesity work by suppressing appetite (such as phentermine) or inhibiting fat absorption (such as orlistat), which can produce serious side effects such as cardiovascular risks, gastrointestinal intolerance, and nutrient malabsorption, thus limiting their long-term use. Recent studies also highlight the limitations of current therapeutic agents, including poor tolerability, rebound weight gain after discontinuation, and lack of efficacy in addressing obesity-associated comorbidities like systemic inflammation and metabolic dysregulation (Bessesen and Van Gaal, 2018; Son and Kim, 2020). While there is a growing interest in glucagon-like peptide 1 (GLP-1) agonists, their high cost, injectable administration, and adverse effects including nausea and pancreatitis restrict widespread clinical adoption (Clemmensen et al., 2019). Thus, there is an urgent need to explore more promising targets and drugs for interventions in obesity (Borah et al., 2021).

Cordycepin (Cpn) is the main bioactive component derived from the medicinal and edible homologous fungus *Cordyceps militaris*, exhibiting diverse pharmacological benefits including antitumor, neuroprotection, immune regulation, and anti-inflammatory properties (Yoon et al., 2018; Tan et al., 2020; Jeong et al., 2010). Early research has shown that Cpn could prevent adipogenesis and lipid accumulation in 3T3-L1 preadipocytes, and decrease the rate of adipocyte differentiation (Liu et al., 2011; Takahashi et al., 2012; Kim et al., 2014). Studies have also unveiled Cpn’s capacity to improve dyslipidemia in hyperlipidemic hamsters and rats, as well as to notably inhibit liver lipid accumulation and reduce liver inflammation in *ob/ob* obese mice (Gao et al., 2011; Zhong et al., 2017). In addition, many studies indicated that Cpn could mitigate body weight and adiposity, and promote the browning of white adipose tissue (WAT) in high-fat diet (HFD)-induced obese mice and rat by modulating adenosine A1 receptor, lipid droplet-encapsulated protein 1, adipose-specific protein 27, adenosine 5′-monophosphate-activated protein kinase (AMPK) or gut microbiota (Xu et al., 2019; Li et al., 2018; Qi et al., 2019; An et al., 2018). Besides, there are studies reported that Cpn intervention could not only regulate lipid metabolism-related proteins and reduce inflammatory responses to improve fatty liver in HFD-induced obese mice but also ameliorate hepatic steatosis in mice with diet-induced nonalcoholic steatohepatitis (Gong et al., 2021; Lan et al., 2021). Furthermore, our previous study has also underscored Cpn’s ability to markedly improve obesity-related metabolic disorders and system inflammation in the Western diet (WD)-induced obese mice by maintaining intestinal microenvironment homeostasis (Chen et al., 2022a). Mechanistically, cordycepin may synergistically combat obesity through multiple pathways, such as activating AMPK to enhance fatty acid oxidation and inhibit lipogenesis, as well as modulating adenosine A1 receptors to regulate adipose tissue browning and thermogenesis. These multimodal actions position Cpn as a potential alternative to existing single-target drugs. Nonetheless, the precise target and regulatory mechanism of Cpn against obesity remain unclear.

Network pharmacology combines bioinformatics, network analysis, and systems biology to illuminate the intricate interactions between drugs, targets, and diseases on a systemic scale (Cai et al., 2019). Recently, network pharmacology-based screening has identified the core targets (PPARγ and AMPK) of oleanolic acid in regulating lipid metabolism, and experimental validation has confirmed its synergistic effects in ameliorating obesity (Liu et al., 2024). This approach serves as a potent tool for pinpointing active constituents and potential targets within traditional Chinese medicine (Jiao et al., 2022), and has been successfully used to reveal the potential mechanisms of action of various natural products (Li et al., 2021; Lv et al., 2021). Transcriptomics, on the other hand, offers a comprehensive analysis of gene transcripts in cells or tissues, unveiling gene expression patterns and regulatory mechanisms across physiological and pathological processes, thereby aiding in the identification of pivotal targets and pathways based on whole genome sequences (Lv et al., 2021). It has been reported in the literature that through transcriptomic analysis, resveratrol (RES) improves obesity through a dual regulatory mechanism involving the AdipoQ signaling axis and lncRNAs related to lipid metabolism (Zhang et al., 2023). In recent years, transcriptomics has proven invaluable in investigating molecular processes and clarifying the protective mechanisms of numerous bioactive compounds (Gu et al., 2022). Hence, integrating transcriptomics with network pharmacology analysis and screening may be an effective strategy for exploring the anti-obesity targets and mechanisms of Cpn.

Thus, this study was designed to screen and unveil the key targets and pathways of Cpn in alleviating obesity in mice by utilizing an integrated strategy that combined network pharmacology, quantitative transcriptomics, molecular docking, and experimental validation. Our findings may offer new insights and substantiate the potential of Cpn as a therapeutic agent for obesity.

## 2 Methods

### 2.1 Materials and reagents

Cordycepin (C805132) with 98% purity was acquired from Shanghai Macklin Biochemical Technology Co., Ltd. (Shanghai, China). The D12079B Western Diet was purchased from Research Diets, Inc. (United States). Hematoxylin (BA-4097) and eosin (BA-4098) staining reagents were obtained from Zhuhai Baso Biotechnology Co., Ltd. (Zhuhai, China). PCR reagents and gDNA Remover (G3337), and qPCR reagents (G3326) were procured from Wuhan Servicebio Technology Co., Ltd. (Wuhan, China). All additional chemicals used were of at least analytical grade.

### 2.2 Animal experiments

The animal experiments followed the National Research Council’s Guide for the Care and Use of Laboratory Animals. The experiments and animal care were carried out in compliance with protocols approved by the Medical Ethics Committee of the First Affiliated Hospital of Guangxi Medical University, with the approval number 2024-E214-01. C57BL/6 J mice (male, 7 weeks old; weighing 20–22 g) were procured from SiPeiFu Biotechnology Co., Ltd. (Beijing, China) and were accommodated in the IVC system under standard conditions.

After 1 week of acclimatization, the mice were randomly assigned to the Chow group (fed with a standard laboratory mice chow diet), the Western diet (WD) group (fed with a WD diet), and the Cpn group (fed with a WD diet and administered with 40 mg/kg Cpn aqueous solution via gavage once daily for 10 weeks). The Cpn dosage was determined according to established literature protocols and validated in our prior dose-response experiments (Qi et al., 2019; Chen et al., 2022b). The Chow and WD group mice received an equivalent volume of distilled water using the same administration method. Each cage housed four mice. Body weight and 24-h food intake were recorded biweekly. At the endpoints of the experiments, mice were fasted overnight and anesthetized by 3% isoflurane. The whole blood samples were collected by retro-orbital puncture. Subsequently, mice were humanely euthanized with an overdose of isoflurane. The liver, perirenal fat, and epididymal fat were harvested and weighed. A portion of the tissue samples was flash-frozen in liquid nitrogen and stored at −80°C for further experiments, while another portion was preserved in a 10% neutral formalin fix solution for histological analyses.

### 2.3 OGTT

After fasting for 16 h, mice were administered 2.0 g/kg glucose (dissolved in saline) by gavage. Blood glucose levels both before and at 30, 60, 90, and 120 min after glucose administration were assessed using a blood glucose meter (Sinocare, GA-7, China) via tail bleeding (Chen et al., 2022a).

### 2.4 Histopathological analysis

Neutral formalin-fixed liver and epididymal adipose tissue were embedded in paraffin and then cut into slices of 5-μm thickness. The slices were stained with hematoxylin and eosin (H&E) (BA-4097, BA-4098, Baso, Zhuhai, China) staining reagents according to the manufacturer’s specifications. Briefly, the slices were deparaffinized to water, and then stained with hematoxylin for approximately 10 min, rinsed with running water, differentiated with hydrochloric acid alcohol, and subsequently rinsed with running water. Following this step, the slices were stained with eosin for 30 s, rinsed again with running water, differentiated with 90% ethanol, and then rinsed with running water. The slices were then subjected to standard dehydration and clarification procedures before being mounted with neutral gum. A scanning microscope (Leica, Aperio CS2, United States) was used to scan the slices and the results were observed with CaseViewer software.

### 2.5 Prediction of anti-obesity targets of cordycepin

The SMLLE name of “cordycepin” and “cordycepin 5′-monophosphate” were obtained from the PubChem database (https://pubchem.ncbi.nlm.nih.gov/). The SMLLE name was inputted into the SwissTargetPrediction database (http://swisstargetprediction.ch/), Similarity Ensemble Approach database (http://sea.bkslab.org/) and PharmMapper Server (http://www.lilab-ecust.cn/pharmmapper/) to identify cordycepin-related targets. Concurrently, the disease-related targets with the keyword “obesity” were searched through the GeneCards database (https://www.genecards.org/), Online Mendelian Inheritance in Man database (http://omim.org/), and Therapeutic Target Database (http://db.idrblab.net/). After removing false positives and duplicates, the cordycepin-related targets were intersected with the obesity-related targets to obtain potential targets for Cpn in treating obesity.

### 2.6 Enrichment analysis

Gene ontology (GO) functional enrichment analysis was performed utilizing the DAVID database (https://david.ncifcrf.gov/) and visualized using the Microbiology Informatics platform (http://www.bioinformatics.com.cn/). The Kyoto Encyclopedia of Genes and Genomics (KEGG) pathway analysis was carried out and visually represented using the Omicshare platform (https://www.omicshare.com/tools/).

### 2.7 Protein-protein interaction network

The protein-protein interaction (PPI) network of potential targets of Cpn was extracted from the STRING database (https://string-db.org/cgi/input.pl/) with a minimum interaction score set at the highest confidence level of 0.4. Parameters such as degree, betweenness, and closeness were acquired using the Centiscape2.2 plug-in. Key nodes relating to the interaction between Cpn, its monophosphate metabolites, and obesity were identified based on meeting or exceeding the median values of all nodes for degree, betweenness, and closeness. The resultant PPI network was then visualized through Cytoscape 3.8.2 software.

### 2.8 Quantitative transcriptomics analysis

Quantitative transcriptomics of epididymal adipose tissue from the three groups of mice was performed by using the nCounter^®^ Metabolic Pathways Panel (XT-CSO-MMP1–12, NanoString Technologie, United States). This panel can detect 768 mRNA transcripts, including 20 internal reference mRNA transcripts. Detailed information about this panel is available at https://www.nanostring.com/products/ncounter-assayspanels/immunology/metabolic-pathway/. The detailed experimental process and data analysis procedures were based on our prior studies (Chen et al., 2022a). Differentially expressed transcripts were screened with a P < 0.05 and visualized using volcano plots. The unsupervised hierarchical clustering heatmaps of the three groups of differentially expressed transcripts were generated and visualized using the Microbiology Informatics platform and TBtools-Ⅱ (Chen et al., 2023) with the complete clustering method. The GO enrichment analysis and KEGG pathway analysis of the differential transcripts were conducted following the procedures outlined in Method 2.6. Subsequently, the overlapping differential transcripts were utilized to construct a PPI network to identify core genes. The methodology employed for constructing the PPI network was consistent with the approach described in Method 2.7.

### 2.9 Molecular docking

Retrieve the InChIKeys for Cordycepin and Cordycepin 5′-monophosphate from the PubChem database and download their respective 2D SDF files. Then search for the three-dimensional crystal structures of proteins within the UniProt database (https://www.uniprot.org/). Import these small molecules and proteins individually into the CB-DOCK2 online molecular docking platform (https://cadd.labshare.cn/cb-dock2/php/index.php). Execute blind docking, record docking scores, and assess these scores post-docking. Select the docking conformation with the lowest score as the optimal configuration. Download the protein-ligand complexes for both molecules with the proteins, and import them into Pymol to create 3D docking interaction diagrams. Finally, transfer these complexes into LigPlus and save them as 2D docking interaction diagrams in PNG format.

### 2.10 Real-time quantitative PCR

Total RNA was extracted from seven randomly selected adipose tissues per group using an RNA extraction solution. RNA concentration and purity were assessed with Nanodrop 2000. Following the manufacturers’ guidelines, reverse transcription was conducted using SweScript All-in-One RT SuperMix and One-Step gDNA Remover. PCR was executed on a CFX Connect detection system (Bio-rad) with 2×Universal Blue SYBR Green qPCR Master Mix. The primers used are listed in Table 1. Each sample was analyzed in triplicate. The results were derived using the 2^−ΔΔCt^ method, with normalization to Gapdh and the Chow group.

**TABLE 1:** Sequences of PCR primers.

Gene	Primer sequence
GAPDH	Forward: 5′-CAG​TGG​CAA​AGT​GGA​GAT​TG-3′
	Reverse: 5′-TGC​CGT​GAG​TGG​AGT​CAT​AC-3′
AKT1	Forward: 5′-AAG​AAG​GAG​GTC​ATC​GTC​GC-3′
	Reverse: 5′-CTT​GAG​GGC​CGT​AAG​GAA​GG-3′
CASP3	Forward: 5′-TGA​GGA​GAT​GGC​TTG​CCA​GA-3′
	Reverse: 5′-TCC​GTT​GCC​ACC​TTC​CTG​TT-3′
EGFR	Forward: 5′-TGA​CTG​TCT​GGT​CTG​CCA​AAA​G-3′
	Reverse: 5′-ATG​CCA​TCT​TCT​TCC​ACT​TCG​T-3′
MAPK14	Forward: 5′-GAC​CGT​TTC​AGT​CCA​TCA​TTC​A-3′
	Reverse: 5′-CTG​GCA​CTT​CAC​GAT​GTT​GTT​C-3′
HRAC	Forward: 5′-CAT​CCA​GCT​GAT​CCA​GAA​CC-3′
	Reverse: 5′-CAT​CTG​AAT​CTT​TCA​CCC​GC-3′
SRC	Forward: 5′-AGA​TCA​CTA​GAC​GGG​AAT​CAG​AGC-3′
	Reverse: 5′-GCA​CCT​TTT​GTG​GTC​TCA​CTC​TC-3′
HSP90AA1	Forward: 5′-TGA​GGA​AAC​CCA​GAC​CCA​AGA-3′
	Reverse: 5′-GCT​GGG​AAT​GAG​ATT​GAT​GTG​C-3′
MMP9	Forward: 5′-GGT​ACT​GGA​AGA​TGT​CGT​GT-3′
	Reverse: 5′-TGA​AGT​CTC​AGA​AGG​TGG​AT-3′
GSK3B	Forward: 5′-TTG​GAG​CCA​CTG​ATT​ACA​CG-3′
	Reverse: 5′-CCA​ACT​GAT​CCA​CAC​CAC​TG-3′
β-Actin	Forward: 5′-GTG​ACG​TTG​ACA​TCC​GTA​AAG​A-3′
	Reverse: 5′-GTA​ACA​GTC​CGC​CTA​GAA​GCA​C-3′

### 2.11 Statistical analysis

The statistical analyses were conducted using GraphPad Prism software (version 9.5.0, San Diego, California, United States). Statistical comparisons between multiple groups were evaluated using one-way ANOVA followed by Dunnett’s multiple comparisons test. The data are presented as the mean ± SEM. A value of P < 0.05 was regarded as statistically significant.

## 3 Results

### 3.1 Cordycepin improved obesity induced by western diet in mice

**FIGURE 1:**
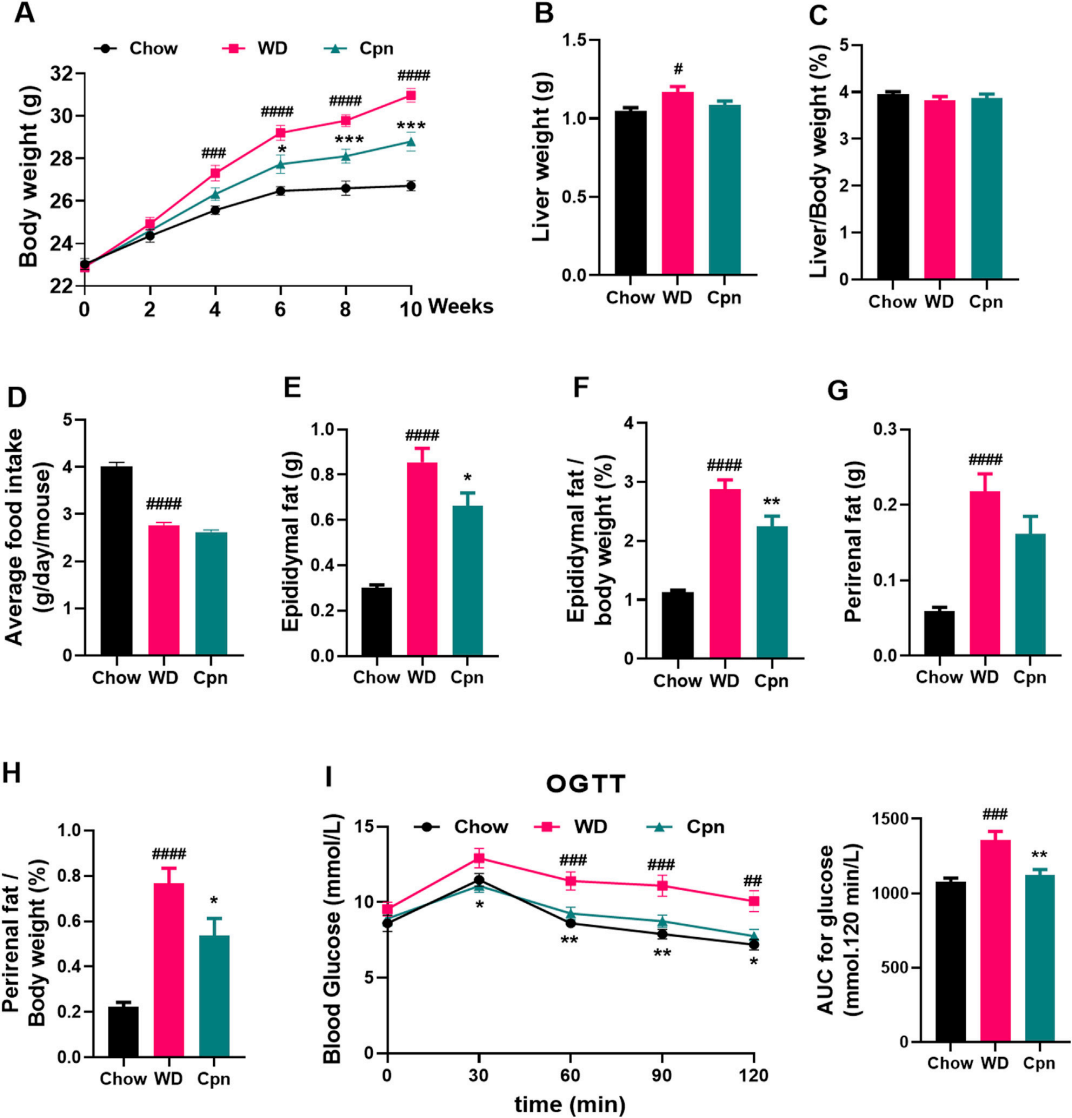
Effect of cordycepin (Cpn) on obesity in the Western diet (WD)-fed mice. **(A)** Body weight changes. **(B)** Liver weight. **(C)** % liver weight of body weight. **(D)** Daily food intake. **(E)** Epididymal fat weight. **(F)** % epididymal fat of total body weight. **(G)** Perirenal fat weight. **(H)** % perirenal fat of total body weight. **(I)** Oral glucose tolerance test. The results are expressed as the mean ± SEM (n = 7–8). P value was determined by one-way ANOVA followed by Dunnett’s test. Where ^#^p < 0.05, ^##^p < 0.01, ^###^p < 0.001 and ^####^p < 0.0001 versus the chow diet (Chow) group; *p < 0.05, **p < 0.01, and ***p < 0.001 versus the WD group.

**FIGURE 2:**
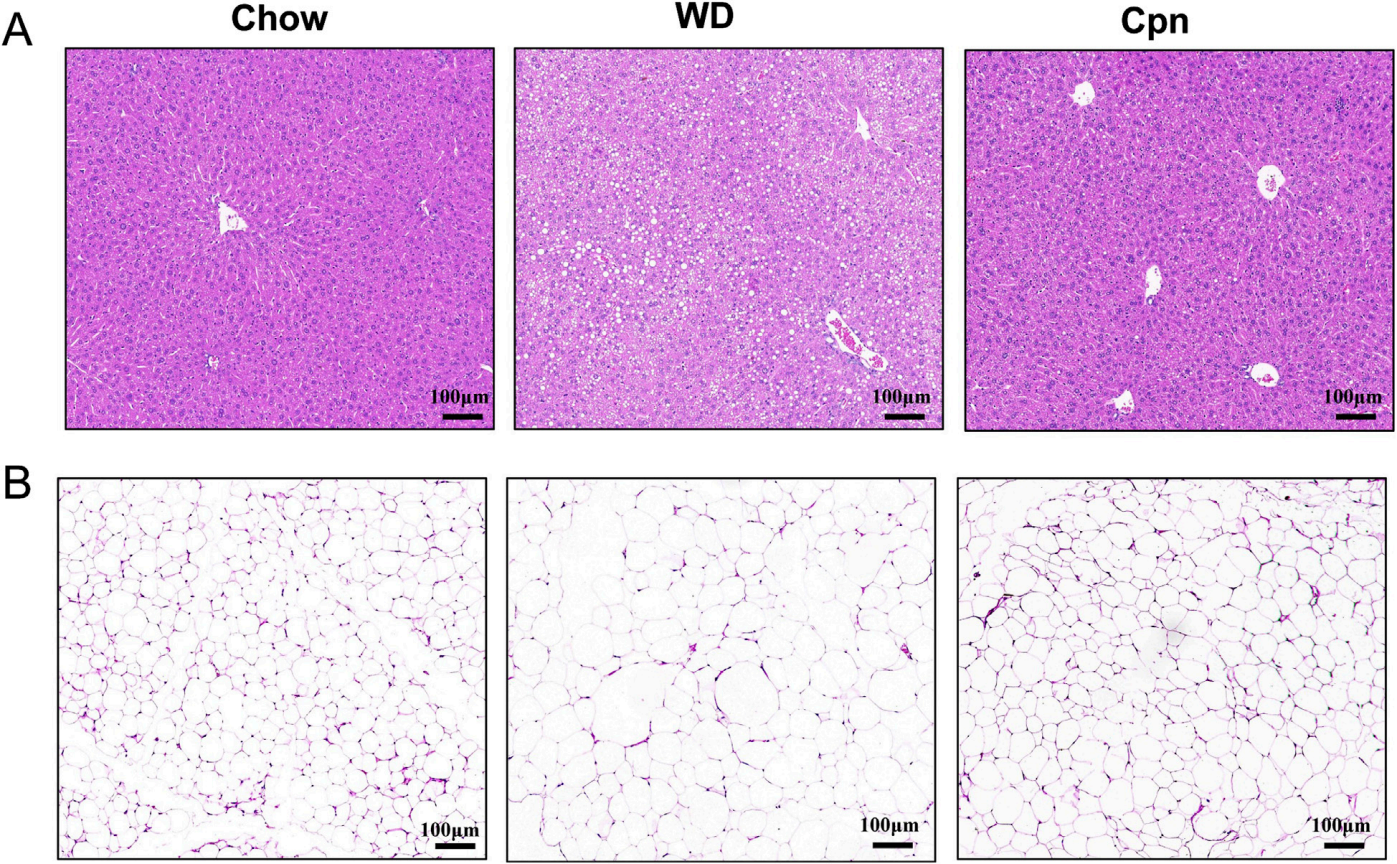
Cordycepin reduced hepatic lipid accumulation and fat cell hypertrophy. Representative images of liver sections **(A)** and epididymal adipose tissue sections **(B)** of different groups stained with H&E. Scale bars, 100 µm.

To investigate the impact of Cpn on obesity, various obesity-related parameters were measured. The results indicated that mice fed a WD for 10 weeks experienced reduced food intake (reduced from 4.01 to 2.76 g/day/mouse) but displayed obvious growth in body weight (increased 15.94%), perirenal fat content (increased 250%), and epididymal fat content (increased 157.1%), coupled with impaired glucose tolerance (decreased 26.03%), compared to those on a standard Chow diet (Figures 1A–H). Mice treated with Cpn showed no change in food intake (2.62 g/day/mouse) but a 8% decrease in body weight compared to mice in the WD group (Figures 1A,D). The liver index of the WD group is 3.83%, and the liver index of the Cpn group is 3.87%. There was no significant change in liver weight, but Cpn treatment reduced 21.88% epididymal fat content and 29.87% perirenal fat content (Figures 1E–H). Additionally, mice treated with Cpn enhanced 17.24% glucose tolerance compared to WD-fed mice (Figure 1I). Moreover, histological examination through H&E staining of liver tissue and epididymal adipose tissue sections revealed an increased number of lipid vacuoles and hypertrophy of adipocytes in the WD group mice. In contrast, the Cpn-treated group had a reduced number of lipid vacuoles and hypertrophy of adipocytes as compared to the WD group (Figure 2). These results supported the effectiveness of Cpn against obesity, consistent with findings from previous studies (Li et al., 2018; Qi et al., 2019; Chen et al., 2022a).

### 3.2 Predicting anti-obesity targets for cordycepin via network pharmacology

#### 3.2.1 Identifying putative cordycepin targets for obesity treatment

**FIGURE 3:**
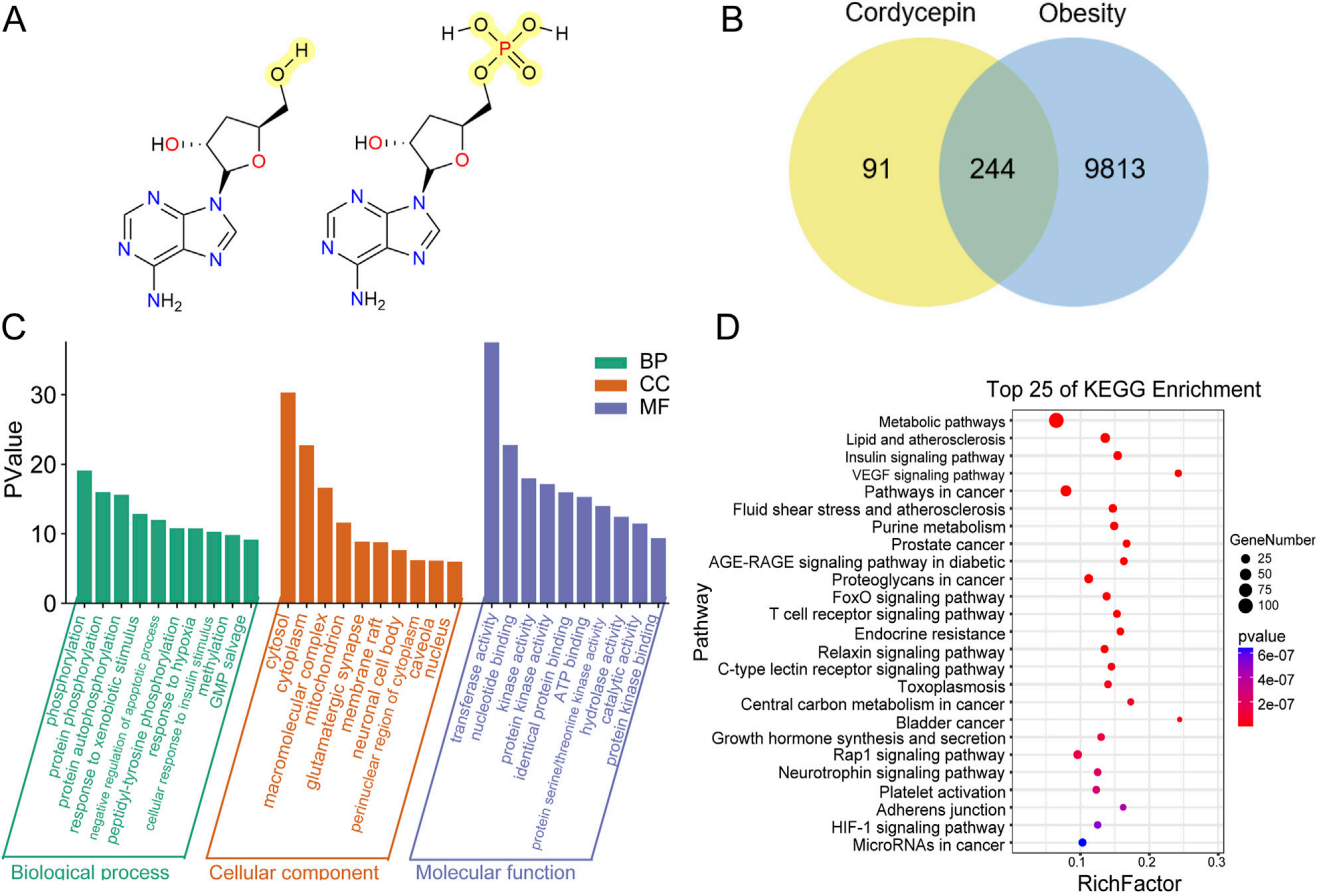
Network pharmacology analysis of cordycepin (Cpn) for anti-obesity. **(A)** Chemical structures of Cpn and cordycepin 5′-monophosphate. **(B)** Venn diagram of Cpn and obesity-related targets. **(C)** The GO functional enrichment analysis and **(D)** KEGG pathway analysis of the potential targets of Cpn against obesity. Pathway and Rich factor are displayed on the Y-axis and X-axis, respectively. The color change represents the P-value. Bubble size indicates the number of genes enriched in the pathway.

**FIGURE 4:**
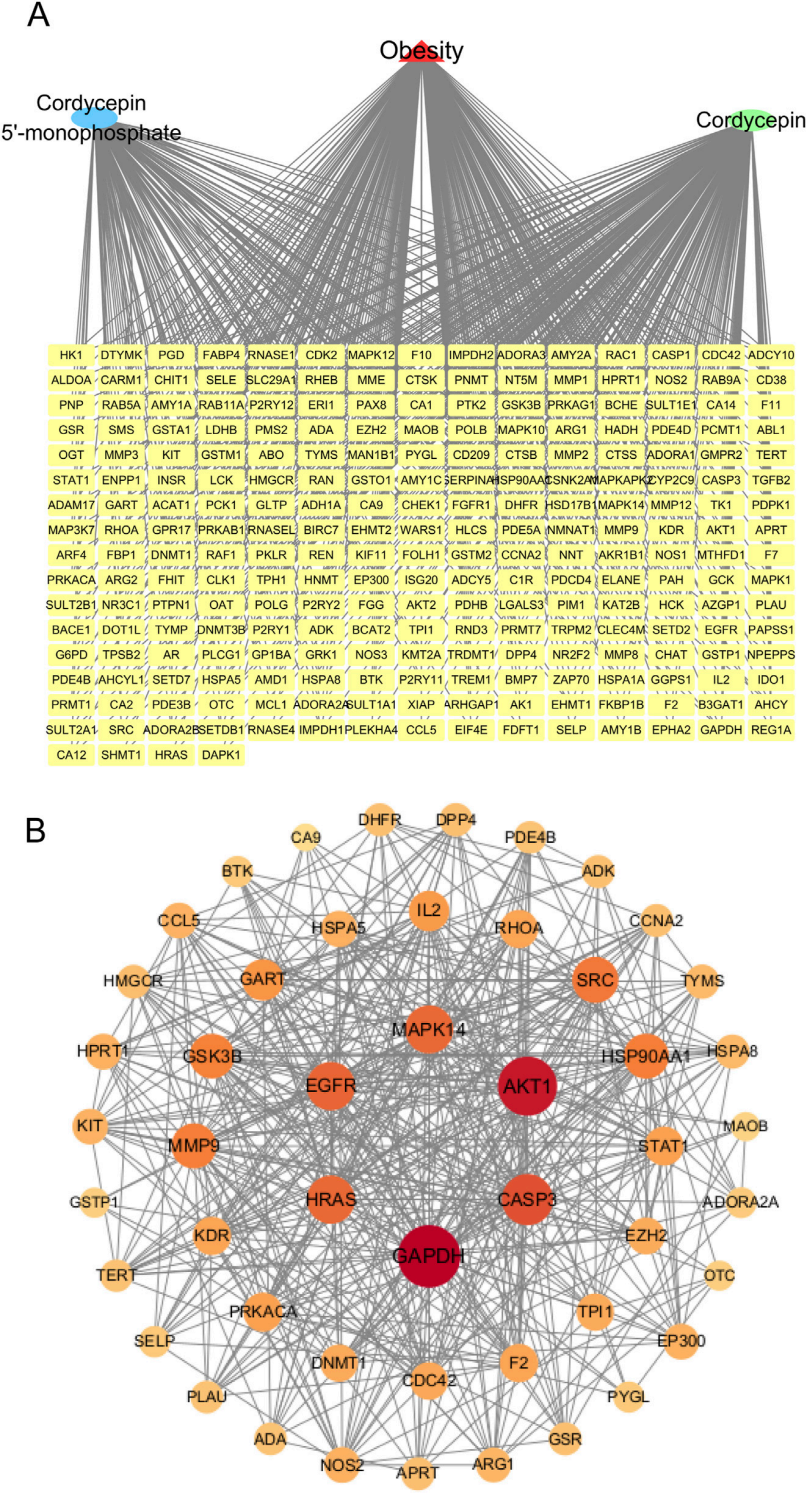
**(A)** A component-target-disease interaction network of Cpn’s role in obesity. **(B)** Protein-protein interaction network of the potential targets of Cpn against obesity. Nodes represent targets of Cpn against obesity, while edges represent target interactions. The larger and darker the red circle, the higher the degree and importance of the target.

To identify potential anti-obesity targets of Cpn, a network pharmacology analysis was conducted. Cordycepin monophosphate, its main active metabolite (Hawley et al., 2020), was also included in the analysis (Figure 3A). Using SwissTargetPrediction, Similarity Ensemble Approach, and PharmMapper Server Databases, we identified 131, 89, and 242 drug targets respectively for Cpn and cordycepin monophosphate. After merging and removing 126 duplicate entries, 335 unique Cpn-related targets remained. Obesity-related targets from the Genecards, OMIM, and Therapeutic Target Database numbered 9,965, 89, and 89 respectively; redundancy reduction yielded a total of 10,057 targets. Intersection of the Cpn targets with these obesity-related targets using a Venn tool identified 244 overlapping targets as candidates for Cpn intervention in obesity (Figure 3B, Supplementary Table S1). A compound-target-disease network was constructed with Cytoscape, illustrating the interactions among Cpn, cordycepin monophosphate, 244 overlapping targets, and obesity. This network consists of 247 nodes and 615 edges, blue and green ellipses denote Cpn and cordycepin monophosphate, yellow rectangles indicate the potential targets of Cpn against obesity and the red triangle represents the disease, obesity (Figure 4A).

#### 3.2.2 GO functional enrichment and KEGG pathway analysis of cordycepin targets

GO functional enrichment analyses were conducted to clarify the biological functions of the 244 potential targets. A total of 587 biological process (BP)-related items, 76 cellular component (CC)-related items, and 160 molecular function (MF)-related items were obtained. The top 10 markedly enriched terms in BP include phosphorylation, protein negative regulation of apoptotic process, response to hypoxia, and cellular response to insulin stimulus. In CC, the main enrichments were in the cytosol, cytoplasm, macromolecular complex, and mitochondrion. MF categories primarily included transferase activity, nucleotide binding, protein kinase activity, and identical protein binding, as shown in Figure 3C.

In addition, a KEGG pathway analysis of these potential targets identified 304 relevant pathways. The top 25 enriched pathways, shown in Figure 3D, included the metabolic pathways, lipid and atherosclerosis, insulin signaling pathway, AGE-RAGE signaling pathway in diabetic complications, and forkhead box O (FOXO) signaling pathway. Notably, pathways like the T cell receptor signaling pathway, chemokine signaling pathway, IL-17 signaling pathway, TNF signaling pathway, and Toll-like receptor signaling pathway were also notably enriched, suggesting that Cpn may ameliorate obesity through multiple pathways, related to both metabolic regulation and inflammatory immunomodulation.

#### 3.2.3 PPI network construction

To identify the essential genes of Cpn against obesity, the 244 potential targets were imported into the STRING database to construct a PPI network. Isolated nodes and low-confidence interactions were excluded, resulting in a network comprising 49 nodes and 452 edges, providing a global view of Cpn’s anti-obesity targets, as shown in Figure 4B. The degree of nodes was an important topological parameter used to assess the importance of targets. Those high-degree nodes in these networks share more target interactions and may exert a more crucial role in anti-obesity. Using the Centiscape2.2 plug-in, the top ten hub genes identified were glyceraldehyde-3-phosphate dehydrogenase (GAPDH), AKT serine/threonine kinase 1 (AKT1), caspase 3 (CASP3), epidermal growth factor receptor (EGFR), mitogen-activated protein kinase 14 (MAPK14), Harvey rat sarcoma viral oncogene homolog (HRAS), proto-oncogene tyrosine-protein kinase Src (SRC), heat shock protein 90 alpha family class A member 1 (HSP90AA1), matrix metalloproteinase 9 (MMP9), and glycogen synthase kinase 3 beta (GSK3B) (Figure 4B; Table 2). These genes may play pivotal roles in the anti-obesity effects of Cpn. Notably, AKT1, MAPK14, SRC, HSP90AA1, and MMP9 have been known to regulate the metabolic pathways and lipid and atherosclerosis pathways mentioned above.

**TABLE 2:** The top 10 nodes with the highest degree in the cordycepin targets’ PPI network.

Target	Degree	Betweenness	Closeness
GAPDH	113	8210.05482	0.00289
AKT1	103	5256.11898	0.00279
CASP3	80	2270.26525	0.00260
EGFR	72	1874.92375	0.00254
MAPK14	71	1580.35899	0.00249
HRAS	71	1596.18739	0.00248
SRC	65	1380.70119	0.00243
HSP90AA1	62	1337.69478	0.0024
MMP9	62	1180.45841	0.00236
GSK3B	61	1126.20683	0.00239

### 3.3 Analysis of cordycepin’s anti-obesity targets using quantitative transcriptomics

**FIGURE 5:**
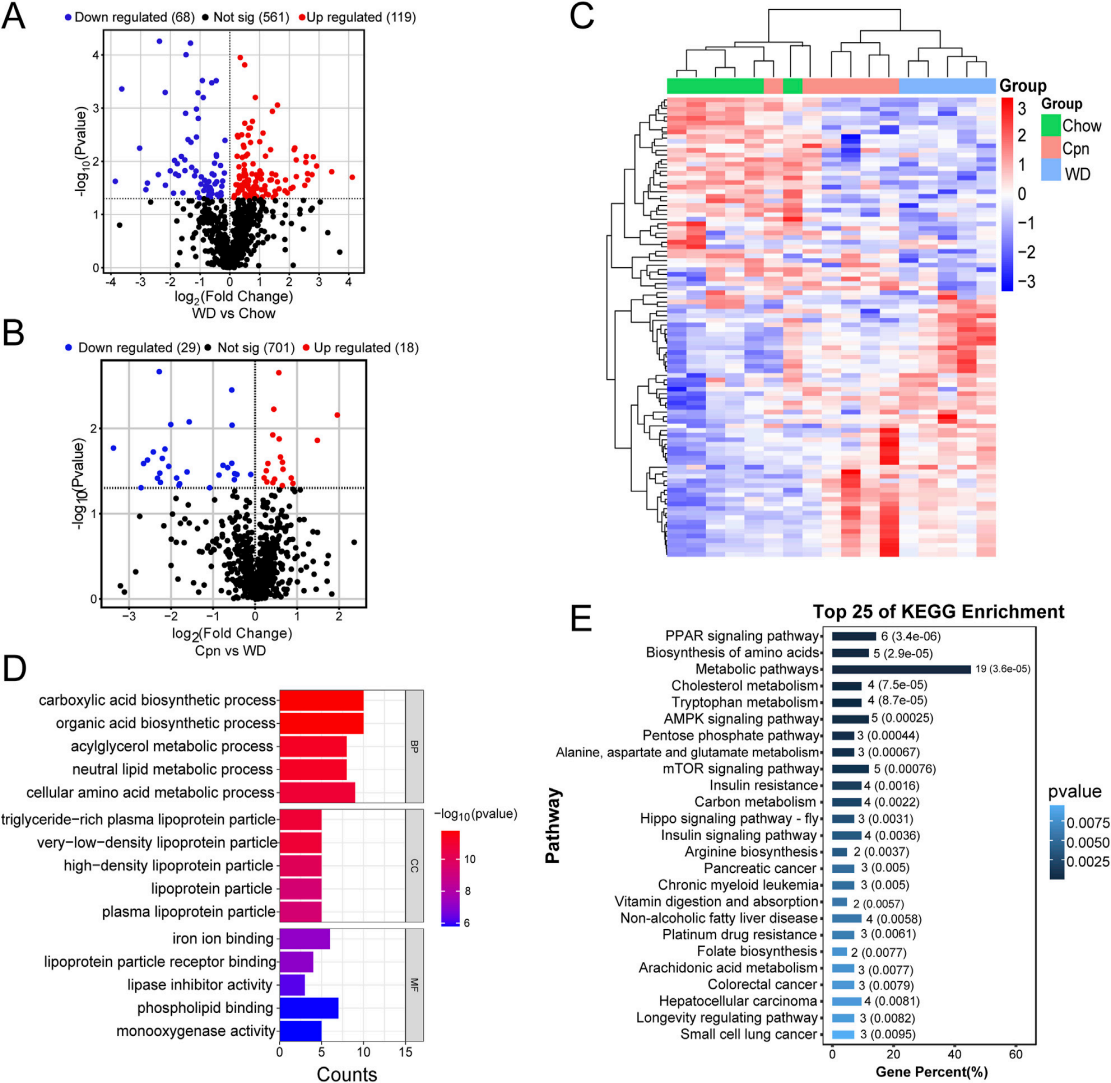
Differential genes analysis of cordycepin (Cpn) based on quantitative transcriptomics. **(A)** Volcano plot of differential genes in the Western diet (WD) and chow diet (Chow) groups (p < 0.05). **(B)** Volcano plot of differential genes in Cpn and WD groups (p < 0.05). **(C)** Heatmap displaying the top 100 genes differentially expressed among the three groups. Upregulated genes are shown in red and downregulated genes in blue. **(D)** GO enrichment analysis and **(E)** KEGG pathway analysis of differential genes derived from the Cpn and WD groups.

To validate the predictions from network pharmacology analysis and expand target screening, quantitative transcriptomics was employed to detect changes in transcripts influenced by Cpn administration in the epididymal adipose tissue of obese mice. Initially, differentially expressed mRNA transcripts were identified and visualized using volcano plots. The comparison between the WD group and the Chow group revealed 187 differentially expressed mRNA transcripts (p < 0.05), with 119 upregulated and 68 downregulated (Figure 5A). In addition, 47 transcripts were found to be differentially expressed (p < 0.05) in the comparison between the Cpn group and the WD group, with 18 upregulated and 29 downregulated (Figure 5B). Further details of these transcripts are provided in Supplementary Tables S2, S3. As illustrated in Figure 5C, distinct mRNA expression profiles were observed across the groups, with Cpn-treated mice showing a closer alignment with Chow-fed mice than WD-fed mice.

Next, GO enrichment analysis was used to determine the biological functions of these differentially expressed genes, to gain a comprehensive understanding of Cpn’s functional role. The analysis revealed that the genes differentially expressed between the WD group and the Chow group were mainly involved in processes such as the tricarboxylic acid cycle, lipid metabolic process, response to oxidative stress, mitochondrion, mitochondrial inner membrane, mitochondrial matrix, identical protein binding, oxidoreductase activity, etc. (Supplementary Figure S1A). As shown in Figure 5D, genes differentially expressed between the Cpn group and the WD group were primarily associated with the carboxylic acid biosynthetic process, organic acid biosynthetic process, neutral lipid metabolic process, very-low-density lipoprotein particle, high-density lipoprotein particle, iron ion binding and lipoprotein particle receptor binding, and phospholipid binding, etc.

Additionally, KEGG pathway analysis showed that genes differentially expressed between the WD group and the Chow group were predominantly enriched in pathways like metabolic pathways, carbon metabolism, biosynthesis of amino acids, chemical carcinogenesis-reactive oxygen species, non-alcoholic fatty liver disease, thermogenesis, and HIF-1 signaling pathway, etc. (Supplementary Figure S1B). Among the Cpn and WD groups, 48 pathways showed enrichment, with the top 25 including the PPAR signaling pathway, biosynthesis of amino acids, metabolic pathways, cholesterol metabolism, tryptophan metabolism, AMPK signaling pathway, insulin resistance, insulin signaling pathway, non-alcoholic fatty liver disease, arachidonic acid metabolism, etc. (Figure 5E; Supplementary Table S4). Notably, pathways such as metabolic pathways, insulin signaling pathway, HIF-1 signaling pathway, FoxO signaling pathway, and lipid and atherosclerosis pathway, etc., aligned with predictions from network pharmacology. This dual verification through network analysis and quantitative transcriptomics supports their crucial role in Cpn’s anti-obesity effects.

**FIGURE 6:**
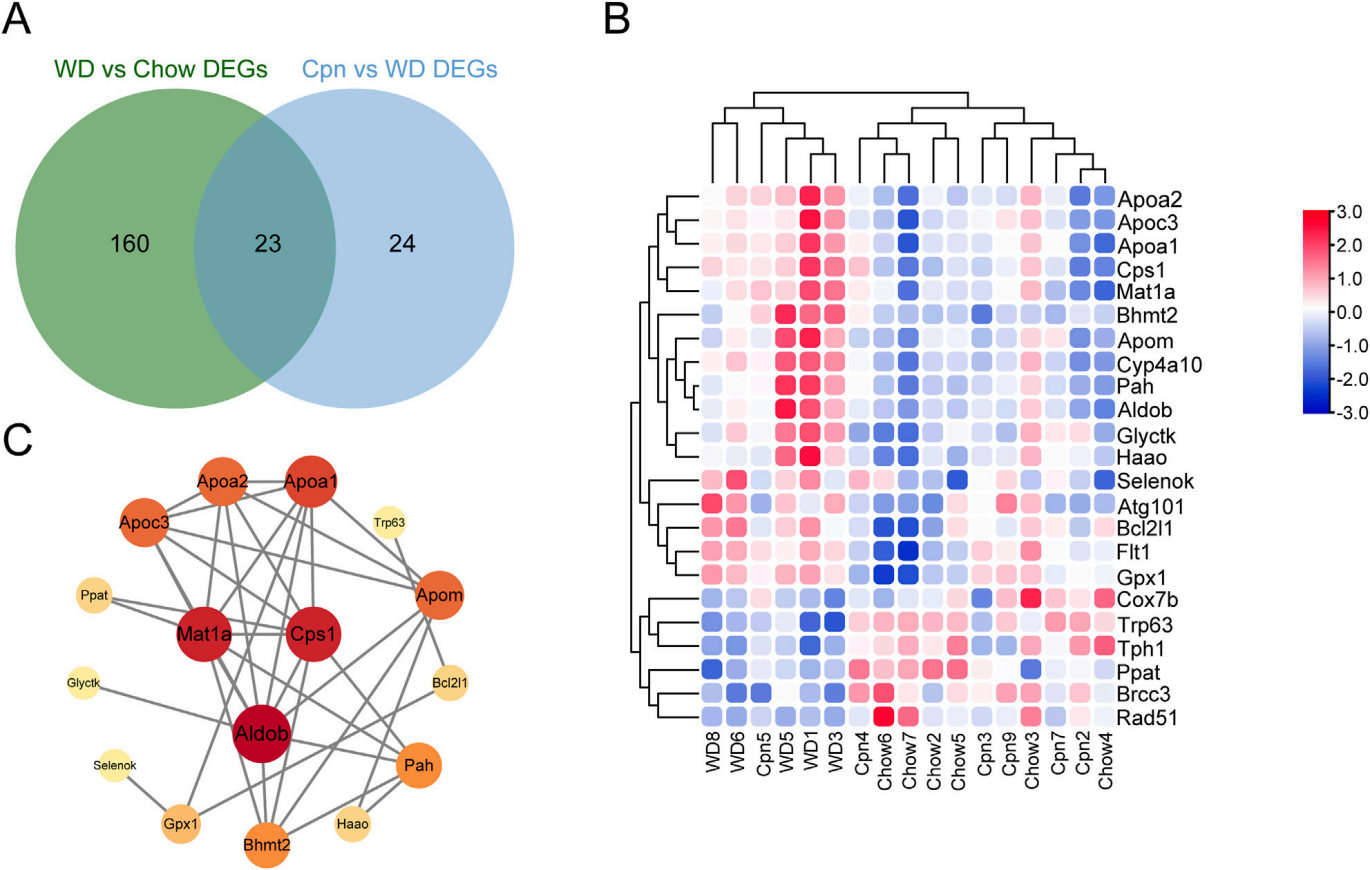
Analysis of potential targets of cordycepin using quantitative transcriptomics. **(A)** Venn diagram of genes (DEGs) differentially expressed among the three groups. **(B)** Heatmap of the 23 genes differential among the three groups. Upregulated genes are shown in red and downregulated genes in blue. **(C)** Protein-protein interaction network of intersecting differential genes.

Furthermore, we hybridized the differential genes of the three groups, and 23 overlapping genes were obtained (Figure 6A). Unsupervised hierarchical clustering of these genes also distinguished the three groups and showed that the Cpn-treated group clustered more closely to the Chow group (Figure 6B). We then constructed a PPI network using these genes and selected hub genes based on their topological characteristics (Figure 6C). Seven genes, including tryptophan 5-hydroxylase 1, cytochrome P450 4A10, DNA repair protein RAD51 homolog 1, lys-63-specific deubiquitinase BRCC36, autophagy-related protein 101, cytochrome c oxidase subunit 7B, and Fms related tyrosine kinase 1, showed no interactions, leaving a network of 16 genes. These 16 genes, detailed in Table 3, had a median degree of connectivity of 4.5, with a maximum of 9, setting the screening criteria for core targets between 4.5 and 9. From this, nine core proteins were identified as potential anti-obesity targets, including fructose-1,6-bisphosphate aldolase b (ALDOB), methionine adenosyltransferase 1A (MAT1A), carbamoyl-phosphate synthase 1 (CPS1), apolipoprotein A1 (APOA1), apolipoprotein C3 (APOC3), apolipoprotein A2 (APOA2), apolipoprotein M (APOM), phenylalanine hydroxylase (PAH), betaine-homocysteine S-methyltransferase 2 (BHMT2).

**TABLE 3:** Node degree in the PPI network of 16 intersection differential genes.

Gene	Degree	Betweenness	Closeness
ALDOB	9	38.03	0.04
MAT1A	8	21.17	0.0385
CPS1	8	21.17	0.0385
APOA1	7	89.2	0.0417
APOC3	6	1.2	0.0357
APOA2	6	1.2	0.0357
APOM	6	20.5	0.0345
PAH	5	9	0.0303
BHMT2	5	1.87	0.0303
GPX1	3	76	0.0313
PPAT	2	0	0.0256
BCL2L1	2	28	0.0227
HAAO	2	0.67	0.0263
SELENOK	1	0	0.0217
TRP63	1	0	0.0172
GLYCTK	1	0	0.0256

Among these nine genes, ALDOB, MAT1A, CPS1, PAH, and BHMT2 were identified as being participated in the biosynthesis of amino acids, and metabolic pathways. Specifically, ALDOB, as a fructose-1,6-bisphosphate aldolase, also involves in the pentose phosphate pathway, carbon metabolism, and HIF-1 signaling pathway. CPS1, functioning as a carbamoyl-phosphate synthase, plays roles in alanine, aspartate, and glutamate metabolism, carbon metabolism, and arginine biosynthesis. APOA1, APOA2, and APOC3, all apolipoproteins, are associated with lipid transport and are notably involved in the PPAR signaling pathway and cholesterol metabolism.

**FIGURE 7:**
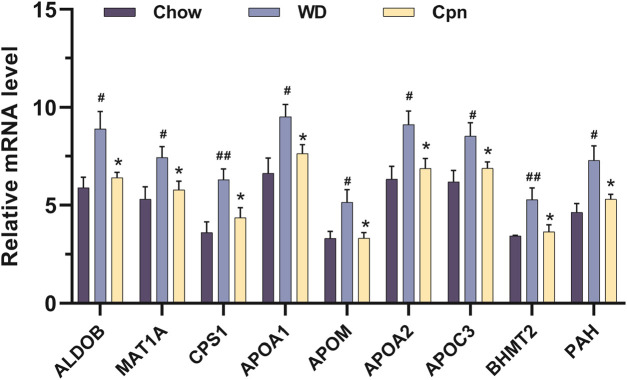
The relative mRNA levels of 9 differential genes among the three groups, normalized against 20 internal references included in the quantitative transcriptomics analysis. n = 5-6 for each group. Where ^#^p < 0.05 and ^##^p < 0.01 versus the chow diet (Chow) group; *p < 0.05 versus the Western diet (WD) group.

Moreover, by analyzing mRNA expression levels, it was found that the expressions of ALDOB, MAT1A, CPS1, APOA1, APOM, APOA2, APOC3, PAH, and BHMT2 were markedly upregulated in the WD group compared to the Chow group. After treatment with Cpn, the expressions of these genes were downregulated (Figure 7). These findings suggested that Cpn’s effectiveness in mitigating obesity may be linked to its regulation of these gene expressions and associated signaling pathways.

### 3.4 Validation of binding capacity between cordycepin and therapeutic targets by molecular docking

**FIGURE 8:**
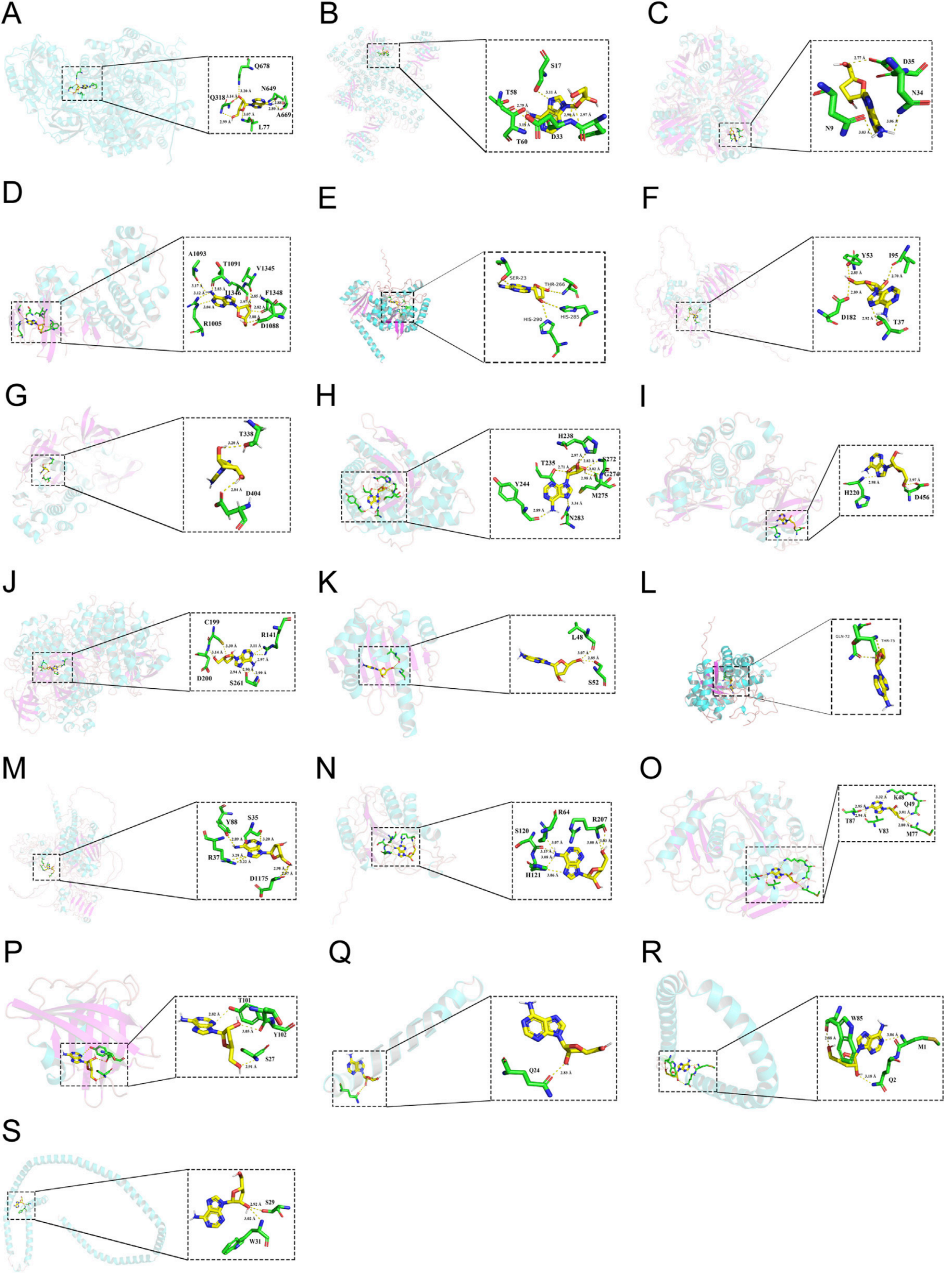
The 3-dimensional map of the binding sites between cordycepin and target proteins. **(A)** CPS1. **(B)** HRAS. **(C)** GAPDH. **(D)** MAPK14. **(E)** PAH. **(F)** MMP9. **(G)** SRC. **(H)** ALDOB. **(I)** AKT1. **(J)** GSK3B. **(K)** HSP90AA1. **(L)** BHMT2. **(M)** EGFR. **(N)** CASP3. **(O)** MAT1A. **(P)** APOM. **(Q)** APOA2. **(R)** APOC3. **(S)** APOA1.

To investigate the potential binding mode and affinity of Cpn with key target proteins, molecular docking was performed on the CB-DOCK2 online docking platform. The docking models with the lowest binding energy were recorded. The findings, detailed in Supplementary Table S5, revealed that Cpn’s highest binding affinity was with CPS1, succeeded by HRAS, GAPDH, MAPK14, PAH, MMP9, SRC, ALDOB, AKT1, GSK3B, HSP90AA1, BHMT2, EGFR, CASP3, MAT1A, APOM, APOA2, APOC3, and APOA1. The specific docking sites of Cpn and target proteins were shown in Figure 8. Among them, Cpn was shown to form hydrogen bonds with specific amino acids in CPS1, including leucine at position 77 and glutamic acid at position 318, asparagine at position 649, alanine at position 669, glutamine at position 678, and exhibited hydrophobic interactions with threonine at position 75, glycine at position 76, glycine at position 78, asparagine at position 319, proline at position 668, phenylalanine at position 677, and glutamate at position 763. Additionally, Cpn 5′-monophosphate may also bind to these proteins, suggesting that it may be the main active metabolite of Cpn in reducing obesity, as shown in Supplementary Figure S2; Supplementary Table S6.

### 3.5 Effect of cordycepin on the potential targets

**FIGURE 9:**
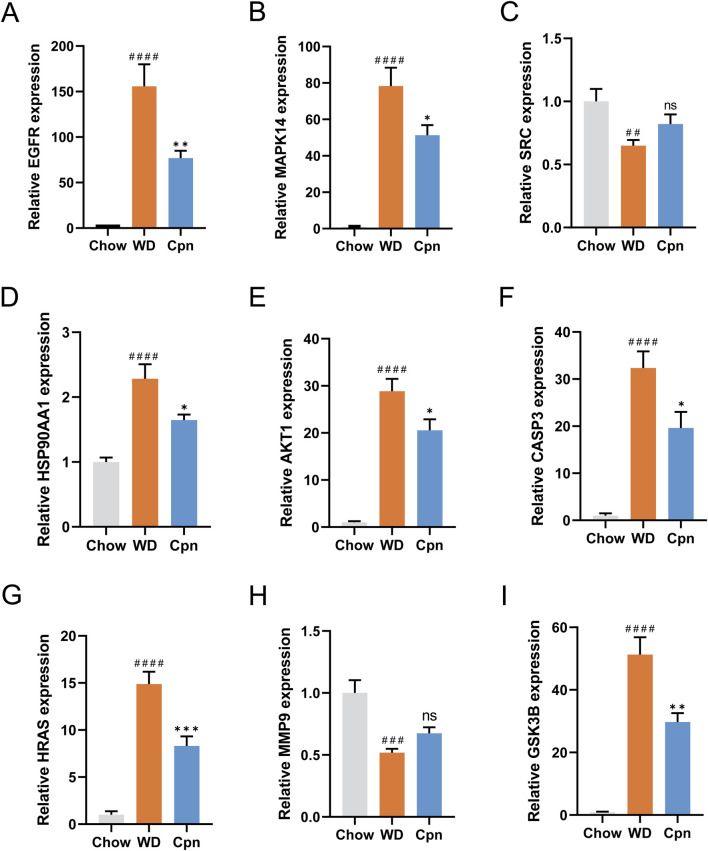
RT-qPCR analysis of the mRNA expression of network pharmacology-predicted core targets. The relative mRNA expression of EGFR **(A)**, MAPK14 **(B)**, SRC **(C)**, HSP90AA1 **(D)**, AKT1 **(E)**, CASP3 **(F)**, HRAS **(G)**, MMP9 **(H)**, and GSK3B **(I)**. The results are expressed as the mean ± SEM (n = 6–7). P value was determined by one-way ANOVA followed by Dunnett’s test. Where ^##^p < 0.01, ^###^p < 0.001 and ^####^p < 0.0001 versus the chow diet (Chow) group; *p < 0.05, **p < 0.01, and ***p < 0.001 versus the Western diet (WD) group.

The regulation of gene expression of ALDOB, MAT1A, CPS1, APOA1, APOM, APOA2, APOC3, PAH, and BHMT2 in epididymal adipose tissue by Cpn was analyzed using quantitative transcriptome data as described above. To further validate this effect on network pharmacology-predicted core targets, mRNA expression levels of these genes were measured in epididymal adipose tissue from the three groups using RT-qPCR. The PCR results indicated that mRNA expression levels of EGFR, MAPK14, HSP90AA1, AKT1, CASP3, HRAS, and GSK3B were obviously elevated in the WD group compared to the Chow group (p < 0.0001), as shown in Figure 9. In contrast, expression levels of SRC and MMP9 were markedly reduced in the WD group compared to the Chow group (p < 0.01). Importantly, administration of Cpn was able to reverse the altered expression of EGFR, MAPK14, HSP90AA1, AKT1, CASP3, HRAS, and GSK3B in the WD group (Figure 9). These findings implied that Cpn’s positive impact on obesity could be due to the modulation of several critical genes.

## 4 Discussion

Obesity has been described as a breeding ground for chronic diseases and a precursor to numerous conditions, such as type 2 diabetes, NAFLD, cardiovascular disease, and cancer. Recent research has increasingly highlighted Cpn’s ability to improve obesity (Takahashi et al., 2012; Li et al., 2018; Qi et al., 2019; Xu et al., 2019). Our earlier investigation demonstrated that Cpn effectively mitigated metabolic irregularities and systemic inflammation induced by a WD in mice (Chen et al., 2022a). In the current research, Cpn was observed to lessen body weight and adiposity, enhance glucose tolerance, and reduce hepatic lipid deposition and adipocyte size in WD-induced obese mice, consistent with previous studies (Takahashi et al., 2012; Li et al., 2018; Qi et al., 2019; Xu et al., 2019). However, the precise regulatory mechanism by which Cpn exerts its anti-obesity properties remains unclear.

In this study, we employed an integrated strategy that combined network pharmacology, quantitative transcriptomics, molecular docking, and experimental validation, to screen and unveil the key targets and pathways of Cpn in alleviating obesity in mice. Initially, we identified the core targets associated with the anti-obesity properties of Cpn through network pharmacology analysis. GO and KEGG enrichment analyses were conducted to elucidate the functions of these crucial targets. Following this, we investigated the key target genes of Cpn in epididymal adipose tissue using quantitative transcriptomics. While the quantitative transcriptomics analysis encompassed only 748 genes, we regrettably did not discover any targets overlapping with network pharmacology insights but instead identified a multitude of novel targets.

The network pharmacology analysis revealed that the anti-obesity targets influenced by Cpn were notably enriched in the metabolic pathways, lipid and atherosclerosis, insulin signaling pathway, AGE-RAGE signaling pathway in diabetic complications, and FoxO signaling pathway, HIF-1 signaling pathway, etc. Moreover, quantitative transcriptomics analysis unveiled that Cpn targets were substantially associated with the PPAR signaling pathway, biosynthesis of amino acids, metabolic pathways, cholesterol metabolism, tryptophan metabolism, AMPK signaling pathway, insulin resistance, insulin signaling pathway, non-alcoholic fatty liver disease, arachidonic acid metabolism, HIF-1 signaling pathway, FoxO signaling pathway, lipid and atherosclerosis pathway, etc.

Interestingly, the metabolic pathway, the insulin signaling pathway, the HIF-1 signaling pathway, the FoxO signaling pathway, and lipid and atherosclerosis pathways emerged as enriched pathways in both the network pharmacology and quantitative transcriptomics analyses. Among them, the metabolic pathway, insulin signaling pathway, and lipid and atherosclerosis pathways have been recognized as crucial regulators in obesity development. Additionally, the HIF-1 signaling pathway and the FoxO signaling pathway are also associated with obesity according to existing literature (Baker et al., 2011; Shi et al., 2023; Peng et al., 2020).

HIF-1α acts as a transcription factor triggered in hypoxic or inflammatory settings, prominently influencing immune inflammation dynamics. Evidence suggests that hypoxia occurs in expanding adipose tissue, and reduced oxygen levels due to diminished perfusion or heightened metabolic rates within adipose tissue may trigger inflammation (Lee et al., 2014). Both rodent studies and clinical observations highlight localized declines in oxygen levels due to obesity (González-Muniesa et al., 2015). Increased HIF-1α levels have been detected within the adipose tissue of obese rodents (Rausch et al., 2008). Through activation of HIF-1α, hypoxia can induce adipocytes to express chemokines such as MCP-1 and LTB4, which may contribute to adipose tissue inflammation (Baker et al., 2011).

FoxO family proteins function as nuclear transcription factors that bind to a specific DNA sequence called the Forkhead box. Within this protein family, FoxO1 has been notably implicated in adipocyte differentiation regulation (Nakae et al., 2003). Studies have highlighted that adipose FoxO1-deficient mice exhibited increased energy expenditure, reduced fat mass, and smaller fat cells (Shi et al., 2023). In addition, Cpn has also been reported to be involved in regulating the expression of FoxO and HIF-1α (Wu et al., 2014; Chang et al., 2019). These findings suggest a potential involvement of these pathways in mediating the anti-obesity effects of Cpn.

Through the construction of PPI networks, with the integration of molecular docking techniques and gene expression validation, we identified numerous potential targets linked to the anti-obesity properties of Cpn. These targets encompass CPS1, HRAS, MAPK14, PAH, ALDOB, AKT1, GSK3B, HSP90AA1, BHMT2 EGFR, CASP3, MAT1A, APOM, APOA2, APOC3, APOA1, etc.

Among these targets, AKT1, MAPK14, and HSP90AA1 have been known to regulate the metabolic pathways and lipid and atherosclerosis pathways mentioned above. ALDOB, MAT1A, CPS1, PAH, and BHMT2 were identified as involved in the metabolic pathways. Specifically, ALDOB also participates in the HIF-1 signaling pathway. APOM, APOA2, APOC3, and APOA1, all apolipoproteins, were associated with lipid transport and involved in the PPAR signaling pathway and cholesterol metabolism, which played important roles in obesity development.

CPS1, the rate-limiting enzyme in the urea cycle, converts toxic ammonia into non-toxic urea in mammals. Recent evidence suggests that CPS1, beyond its tumor-related roles, also influences metabolic diseases. Studies have shown that in high-fat diet (HFD)-induced NAFLD, the gene and protein expression levels of CPS1, involved in hepatic nitrogen conversion, decrease (Luo et al., 2012; Eriksen et al., 2019). Moreover, in fibrosis models induced by a choline-deficient high-fat diet, hepatic CPS1 expression declines with escalating fibrosis levels (Gallego-Durán et al., 2022). Conversely, CPS1 levels are elevated in the plasma of NAFLD patients with varying fibrosis extents and in the serum of individuals experiencing acetaminophen-induced acute liver failure, with levels diminishing post-injury recovery (Ajaz et al., 2021; Kwan et al., 2023). Additionally, reports suggest that activation of AMPK suppresses CPS1 expression (Zhang et al., 2021). Cpn is known to promote the phosphorylation of AMPK protein and activate AMPK. In this study, WD-induced obese mice displayed a notably increase in CPS1 expression in epididymal adipose tissue, while CPS1 expression was reversed after Cpn treatment. Furthermore, molecular docking results showed that Cpn forms hydrogen bonds and hydrophobic interactions with CPS1 protein amino acid residues, indicating potential binding between CPS1 and Cpn. This implies that CPS1 may serve as a crucial target for Cpn against obesity.

HRAS, a member of the RAS oncogene family, plays a pivotal role in cell division, differentiation, and apoptosis. Recent studies have reported its close association with the modulation of energy homeostasis. For instance, HRAS G12S heterozygous mutant mice displayed resistance to HFD-induced obesity but exhibited impaired hepatic energy homeostasis compared to wild-type mice (Oba et al., 2018). HRAS has also been implicated in influencing adipocyte differentiation in 3T3-L1 precursors (Park et al., 2019). Mice with adipose-specific HRAS transgenes exhibited reduced weight and size of gonadal fat pads, enhanced glucose tolerance, and lowered plasma insulin levels (Houseknecht et al., 1996). While these studies underscore HRAS’s involvement in adipocyte differentiation regulation, the precise mechanisms remain unclear. Furthermore, reports indicated that Cpn downregulates HRAS expression in cisplatin-resistant lung cancer cells to exert antiproliferative effects (Cho and Kang, 2018). In the present investigation, we observed that Cpn notably decreased the upregulation of HRAS expression in adipose tissue induced by a WD, suggesting that HRAS may be a potential target for Cpn in alleviating obesity.

The MAPK14 gene encodes mitogen-activated protein kinase 14, also identified as p38α. p38α has been recognized as a key player in activating adipose tissue thermogenesis. Studies have revealed that knocking down p38α specifically in adipocytes promotes WAT browning, leading to a leaner phenotype, enhanced metabolism, and resistance to diet-induced obesity in mice (Zhang et al., 2018). The p38α inhibitor SB203580 exhibits beneficial metabolic effects by enhancing WAT browning (Zhang et al., 2018). Inhibition of p38α triggers UCP-1 transcription, instigating the transition from white to beige fat. Additionally, an HFD boosts p38 activity in adipose-resident T cells in murine models (Meng et al., 2022). Selective deletion of T-cell-specific p38α manifests elevated energy expenditure and protects mice against HFD-induced obesity (Meng et al., 2022). P38α plays a crucial role in adipose thermogenesis, although some research indicates that the impact of p38α on regulating adipose tissue browning can vary based on the specific adipose depot location (Matesanz et al., 2018). Further investigations have revealed that Cpn can inhibit p38 MAPK phosphorylation, showing promising anti-inflammatory effects (Yang et al., 2015; Jeong et al., 2010). Moreover, studies have highlighted that Cpn can enhance the expression of thermogenic genes such as UCP1, and peroxisome proliferator-activated receptor γ coactivator 1α, etc., by activating AMPK, subsequently facilitating WAT browning in HFD-induced obese mice (Qi et al., 2019). Therefore, p38α may be involved in the regulation of obesity by cordycepin.

PAH, which encodes phenylalanine hydroxylase, catalyzes the hydroxylation of phenylalanine to tyrosine, representing the rate-limiting step in phenylalanine catabolism. Many studies have found that phenylalanine is closely related to the occurrence and development of obesity (Liu et al., 2022; Li et al., 2022; Xiao et al., 2024). Chronic administration of N-lactoyl-phenylalanine reduced body weight and adiposity and enhances glucose homeostasis.(Li et al., 2022). In addition, N-lactoyl-phenylalanine has also been established as a crucial mediator of metformin’s effects in reducing body weight (Xiao et al., 2024). Our findings indicated a potential interaction between PAH and Cpn. In addition, the WD significantly induced the gene expression of PAH, which can be reversed by Cpn administration. Therefore, we speculate that PAH may be a critical target for Cpn to reduce obesity.

ALDOB functions as a fructose diphosphate aldolase, catalyzing the breakdown of fructose 1-phosphate into dihydroxyacetone phosphate and glyceraldehyde. Studies by Nesteruk et al. revealed increased hepatic and serum ALDOB in HFD-induced NAFLD mice compared to those on a standard diet (Nesteruk et al., 2014). Similarly, research by Niu et al. and Martinez-Huenchullan et al. observed increased plasma ALDOB protein in both NAFLD patients and mice (Niu et al., 2019; Martinez-Huenchullan et al., 2021). Moreover, hepatic ALDOB expression was notably elevated in *db/db* mice relative to control mice, indicating a potential association with excessive fat accumulation in hepatocytes (Zhang et al., 2022).

Besides, the mRNA levels of ALDOB in pancreatic islets from hyperglycemic patients were negatively correlated with insulin secretion and positively correlated with HbA1c levels, with hyperglycemic conditions augmenting ALDOB expression (Gerst et al., 2018). Our investigation unveiled a significant upregulation of ALDOB expression in epididymal adipose tissue in obese mice induced by a WD (p < 0.05), whereas treatment with Cpn effectively reversed this expression. This observation suggests that Cpn might mitigate obesity in mice by modulating ALDOB expression.

AKT1 also referred to as PKBα, is a pivotal member of the phosphokinase B family, playing a crucial role in the phosphatidylinositol 3-kinase (PI3K)/AKT/mammalian target of the rapamycin signaling pathway. Studies have demonstrated that AKT1 deficiency in mice enhances energy expenditure and prevents diet-induced obesity (Wan et al., 2012). Moreover, inhibition of PI3Kα-AKT1 was reported to induce energy expenditure in adipose tissue, suppress weight gain, and ameliorate insulin resistance in diet-induced obese mice (Song et al., 2018). Beyond its involvement in adipose thermogenesis, AKT1 has been implicated in immune-inflammatory regulation. Specifically, in LPS-stimulated macrophages, the activation of AKT1 expression triggers the NF-κB signaling pathway, leading to the upregulation of pro-inflammatory factors (Yen et al., 2015; Li et al., 2019). Cpn has been identified for its ability to impede preadipocyte differentiation, which was attributed to its interference with the AKT inhibition of mTORC1 and activation of AMPK, thus hindering the lipid-forming CCAAT/enhancer binding protein-β (C/EBPβ)-PPARγ pathway (Takahashi et al., 2012).

Moreover, GSK3B is a pivotal downstream signaling protein in the PI3K/AKT pathway. Insulin-triggered AKT activation via PI3K leads to the serine/threonine phosphorylation of GSK3B, which in turn phosphorylates key targets like C/EBPβ, C/EBPα, and glycogen synthase (Ross et al., 1999). Inhibition of GSK3B through lithium treatment prevented 3T3-L1 preadipocytes from differentiating into adipocytes (Ross et al., 1999). Similarly, GSK3B has been implicated as a major regulator of the inflammatory response. In individuals with metabolic irregularities affecting obesity, GSK3B fosters NF-κB and CREB activation, prompting the expression of inflammatory factors in leukocytes (Yen et al., 2015). Studies have demonstrated that Cpn regulates the phosphorylation level of GSK3B (Khan and Tania, 2023). In our previous study, Cpn can Notably diminish the expression of serum inflammatory factors and alleviate systemic inflammation in mice subjected to a WD. The results of this study indicated that Cpn has a notable impact on the mRNA expression of AKT1 and GSK3B in the epididymal adipose tissue of obese mice, suggesting that AKT1 and GSK3B are potential regulatory targets of Cpn.

Therefore, Cpn may be a promising pharmacological agent for the modulation of the aforementioned therapeutic targets in an integrated approach toward mitigating the development of obesity. The strength of this study is the comprehensive identification of Cpn’s therapeutic targets for alleviating diet-induced obesity. This was achieved through network pharmacology analysis integrating multiple databases, precise quantitative sequencing analysis with Nanostring nCounter technology, and validation of identified targets through molecular docking simulations and mRNA-level tissue expression experiments. Numerous studies have explored the use of Cpn in mitigating obesity, yet no published study has undertaken such an extensive screening and validation of therapeutic targets for Cpn against obesity. These results have provided a detailed understanding of the potential therapeutic targets of Cpn in the context of obesity and lay a foundational framework for its application in obesity treatment.

Nonetheless, this study has some limitations. This study focuses on the research of epididymal adipose tissue, but Cpn may not specifically act on adipose tissue. Although many studies have reported that Cpn can reduce fat content, promote thermogenesis in adipose tissue, and inhibit the differentiation of 3T3-L1 cells (Xu et al., 2019; Li et al., 2018; Qi et al., 2019; An et al., 2018), there are also studies that report its role in improving non-alcoholic fatty liver disease (Gong et al., 2021; Lan et al., 2021). In addition, our preliminary research has also found that Cpn improved metabolic inflammation by acting on the colon and intestinal flora, and metabolic inflammation has been considered a key driver of obesity (Chen et al., 2022a). Therefore, this study cannot rule out the role of Cpn in improving obesity through crosstalk between the liver and intestines and even other tissues and organs, but it is certain that Cpn has a regulatory effect on adipocytes and adipose tissue. As for the crosstalk effects of other tissues and organs, further research is needed.

At present, we have identified numerous relevant targets. In the future, gene editing technology will be applied to knock out/knock in potential targets to verify their necessity in the anti-obesity effect of Cpn. Additionally, like most natural products, Cpn has the issue of low bioavailability. We expect to enhance its bioavailability and the ability to target adipose tissue by designing nanocarriers (such as liposomes, exosomes) or prodrug modifications, or by developing Cpn derivatives through chemical modifications to increase targeting affinity or reduce toxicity. Cpn originates from the edible and medicinal fungus *Cordyceps sinensis*. In the future, it is possible to develop functional foods or precise nutrition intervention plans based on Cpn. Furthermore, existing anti-obesity drugs (such as GLP-1 receptor agonists) or lifestyle interventions (such as exercise, dietary regulation) could be considered in combination to assess the synergistic effect.

## 5 Conclusion

Despite the aforementioned limitations, we concluded that therapeutic pathways and targets for Cpn against obesity can be comprehensively screened through network pharmacology, precise quantitative transcriptomics, bioinformatics analysis, molecular docking simulation, and *in vivo* validation. Our results indicated that Cpn treatment effectively alleviated obesity-related symptoms in WD-induced mice. The metabolic pathway, insulin signaling pathway, HIF-1 signaling pathway, FoxO signaling pathway, lipid and atherosclerosis pathway, and core targets including CPS1, HRAS, MAPK14, PAH, ALDOB, AKT1, GSK3B, HSP90AA1, BHMT2, EGFR, CASP3, MAT1A, APOM, APOA2, APOC3, and APOA1 are involved in regulating the therapeutic effect of Cpn. This study comprehensively revealed the potential mechanism for Cpn against obesity and supplied supporting data for its efficacy in treating obesity and its associated complications.

## Data Availability

The datasets presented in this study can be found in online repositories. The names of the repository/repositories and accession number(s) can be found in the article/Supplementary Material.

## Glossary

Cpn: Cordycepin
WD: Western diet
NAFLD: Non-alcoholic fatty liver disease
AMPK: Activating adenosine 5′-monophosphate-activated protein kinase
OGTT: Oral glucose tolerance tests
H&E: Hematoxylin and eosin
GO: Gene ontology
BP: Biological process
CC: Cellular component
MF: Molecular function
KEGG: The Kyoto Encyclopedia of Genes and Genomics
PPI: Protein-protein interaction
GAPDH: Glyceraldehyde-3-phosphate dehydrogenase
AKT1: AKT serine/threonine kinase 1
CASP3: Caspase 3
EGFR: Epidermal growth factor receptor
MAPK14: Mitogen-activated protein kinase 14
HRAS: Harvey rat sarcoma viral oncogene homolog
SRC: Proto-oncogene tyrosine-protein kinase Src
HSP90AA1: Heat shock protein 90 alpha family class A member 1
MMP9: Matrix metalloproteinase 9
GSK3B: Glycogen synthase kinase 3 beta
HIF-1: Hypoxia-inducible factor 1
FoxO: Forkhead box O
AGE-RAGE: Advanced glycation end products-Receptor for advanced glycation end products
PPAR: Peroxisome proliferator-activated receptors
ALDOB: Fructose-1,6-bisphosphate aldolase b
MAT1A: Methionine adenosyltransferase 1A
CPS1: Carbamoyl-phosphate synthase 1
APOA1: Apolipoprotein A1
APOC3: Apolipoprotein C3
APOA2: Apolipoprotein A2
APOM: Apolipoprotein M
PAH: Phenylalanine hydroxylase
BHMT2: Betaine-homocysteine S-methyltransferase 2
UCP1: Uncoupling protein 1
WAT: White adipose tissue
CREB: cAMP-responsive element-binding protein
PKB: Phosphokinase B
PI3K: Phosphatidylinositol 3-kinase
C/EBPβ: CCAAT/enhancer binding protein-β
NF-κB: Nuclear factor kappa-B

## References

[B1] Ajaz S. Mcphail M. J. Gnudi L. Trovato F. M. Mujib S. Napoli S. (2021). Mitochondrial dysfunction as a mechanistic biomarker in patients with non-alcoholic fatty liver disease (NAFLD). Mitochondrion 57, 119–130. 10.1016/j.mito.2020.12.010 33387664

[B2] An Y. Li Y. Wang X. Chen Z. Xu H. Wu L. (2018). Cordycepin reduces weight through regulating gut microbiota in high-fat diet-induced obese rats. Lipids Health Dis. 17, 276. 10.1186/s12944-018-0910-6 30522511 PMC6284310

[B3] Baker R. G. Hayden M. S. Ghosh S. (2011). NF-κB, inflammation, and metabolic disease. Cell Metab. 13, 11–22. 10.1016/j.cmet.2010.12.008 21195345 PMC3040418

[B4] Bessesen D. H. Gaal L. F. (2018). Progress and challenges in anti-obesity pharmacotherapy. Lancet Diabetes and Endocrinol. 6, 237–248. 10.1016/S2213-8587(17)30236-X 28919062

[B5] Borah A. K. Sharma P. Singh A. Kalita K. J. Saha S. Chandra Borah J. (2021). Adipose and non-adipose perspectives of plant derived natural compounds for mitigation of obesity. J. Ethnopharmacol. 280, 114410. 10.1016/j.jep.2021.114410 34273447

[B6] Cai F. F. Bian Y. Q. Wu R. Sun Y. Chen X. L. Yang M. D. (2019). Yinchenhao decoction suppresses rat liver fibrosis involved in an apoptosis regulation mechanism based on network pharmacology and transcriptomic analysis. Biomed. Pharmacother. 114, 108863. 10.1016/j.biopha.2019.108863 30991286

[B7] Chang M. M. Pan B. S. Wang C. Y. Huang B. M. (2019). Cordycepin-induced unfolded protein response-dependent cell death, and AKT/MAPK-mediated drug resistance in mouse testicular tumor cells. Cancer Med. 8, 3949–3964. 10.1002/cam4.2285 31145545 PMC6639181

[B8] Chen J. M. Wang M. C. Zhang P. Li H. Qu K. Xu R. M. (2022a). Cordycepin alleviated metabolic inflammation in Western diet-fed mice by targeting intestinal barrier integrity and intestinal flora. Pharmacol. Res. 178, 106191. 10.1016/j.phrs.2022.106191 35346845

[B9] Chen C. Wu Y. Li J. Wang X. Zeng Z. Xu J. (2023). TBtools-II: a “one for all, all for one” bioinformatics platform for biological big-data mining. Mol. Plant 16, 1733–1742. 10.1016/j.molp.2023.09.010 37740491

[B10] Chen J. Wang M. Zhang P. Li H. Qu K. Xu R. (2022b). Cordycepin alleviated metabolic inflammation in Western diet-fed mice by targeting intestinal barrier integrity and intestinal flora. Pharmacol. Res., 178. 10.1016/j.phrs.2022.106191 35346845

[B11] Cho S. H. Kang I. C. (2018). The inhibitory effect of Cordycepin on the proliferation of cisplatin-resistant A549 lung cancer cells. Biochem. Biophys. Res. Commun. 498, 431–436. 10.1016/j.bbrc.2018.02.188 29496448

[B12] Clemmensen C. Finan B. Muller T. D. Dimarchi R. D. Tschop M. H. Hofmann S. M. (2019). Emerging hormonal-based combination pharmacotherapies for the treatment of metabolic diseases. Nat. Rev. Endocrinol. 15, 90–104. 10.1038/s41574-018-0118-x 30446744

[B13] De A. B. A. P. De O. C. P. H. Be F. F. Ps E. S. De Moraes L. Migliolo L. (2023). Adipose tissue, systematic inflammation, and neurodegenerative diseases. Neural Regen. Res. 18, 38–46. 10.4103/1673-5374.343891 35799506 PMC9241402

[B14] Eriksen P. L. Vilstrup H. Rigbolt K. Suppli M. P. SøRENSEN M. HeebøLL S. (2019). Non-alcoholic fatty liver disease alters expression of genes governing hepatic nitrogen conversion. Liver Int. 39, 2094–2101. 10.1111/liv.14205 31386258

[B15] Gallego-DuráN R. Ampuero J. Pastor-RamíREZ H. Álvarez-Amor L. Del Campo J. A. Maya-Miles D. (2022). Liver injury in non-alcoholic fatty liver disease is associated with urea cycle enzyme dysregulation. Sci. Rep. 12, 3418. 10.1038/s41598-022-06614-9 35232986 PMC8888708

[B16] Gao J. Lian Z. Q. Zhu P. Zhu H. B. (2011). Lipid-lowering effect of cordycepin (3'-deoxyadenosine) from Cordyceps militaris on hyperlipidemic hamsters and rats. Yao Xue Xue Bao 46, 669–676. 21882527

[B17] Gerst F. Jaghutriz B. A. Staiger H. Schulte A. M. Lorza-Gil E. Kaiser G. (2018). The expression of aldolase B in islets is negatively associated with insulin secretion in humans. J. Clin. Endocrinol. Metab. 103, 4373–4383. 10.1210/jc.2018-00791 30202879 PMC6915830

[B18] Gong X. Li T. Wan R. Sha L. (2021). Cordycepin attenuates high-fat diet-induced non-alcoholic fatty liver disease via down-regulation of lipid metabolism and inflammatory responses. Int. Immunopharmacol. 91, 107173. 10.1016/j.intimp.2020.107173 33352441

[B19] GonzáLEZ-Muniesa P. Garcia-Gerique L. Quintero P. Arriaza S. Lopez-Pascual A. Martinez J. A. (2015). Effects of hyperoxia on oxygen-related inflammation with a focus on obesity. Oxid. Med. Cell Longev. 2015, 8957827. 10.1155/2016/8957827 26697142 PMC4678090

[B20] Gu Y. Bai J. Zhang J. Zhao Y. Pan R. Dong Y. (2022). Transcriptomics reveals the anti-obesity mechanism of Lactobacillus plantarum fermented barley extract. Food Res. Int. 157, 111285. 10.1016/j.foodres.2022.111285 35761593

[B21] Hawley S. A. Ross F. A. Russell F. M. Atrih A. Lamont D. J. Hardie D. G. (2020). Mechanism of activation of AMPK by cordycepin. Cell Chem. Biol. 27, 214–222 e4. 10.1016/j.chembiol.2020.01.004 31991096 PMC7031697

[B22] Houseknecht K. L. Zhu A. X. Gnudi L. Hamann A. Zierath J. R. Tozzo E. (1996). Overexpression of Ha-ras selectively in adipose tissue of transgenic mice. Evidence for enhanced sensitivity to insulin. J. Biol. Chem. 271, 11347–11355. 10.1074/jbc.271.19.11347 8626688

[B23] Jeong J. W. Jin C. Y. Kim G. Y. Lee J. D. Park C. Kim G. D. (2010). Anti-inflammatory effects of cordycepin via suppression of inflammatory mediators in BV2 microglial cells. Int. Immunopharmacol. 10, 1580–1586. 10.1016/j.intimp.2010.09.011 20937401

[B24] Jiao W. Mi S. Sang Y. Jin Q. Chitrakar B. Wang X. (2022). Integrated network pharmacology and cellular assay for the investigation of an anti-obesity effect of 6-shogaol. Food Chem. 374, 131755. 10.1016/j.foodchem.2021.131755 34883426

[B25] Khan M. A. Tania M. (2023). Cordycepin and kinase inhibition in cancer. Drug Discov. Today 28, 103481. 10.1016/j.drudis.2022.103481 36584876

[B26] Kim S. B. Ahn B. Kim M. Ji H. J. Shin S. K. Hong I. P. (2014). Effect of Cordyceps militaris extract and active constituents on metabolic parameters of obesity induced by high-fat diet in C58BL/6J mice. J. Ethnopharmacol. 151, 478–484. 10.1016/j.jep.2013.10.064 24231073

[B27] Kwan R. Chen L. Park M. J. Su Z. Weerasinghe S. V. W. Lee W. M. (2023). The role of carbamoyl phosphate synthetase 1 as a prognostic biomarker in patients with acetaminophen-induced acute liver failure. Clin. Gastroenterol. Hepatol. 21, 3060–3069.e8. 10.1016/j.cgh.2023.03.002 37054752 PMC10656042

[B28] Lan T. Yu Y. Zhang J. Li H. Weng Q. Jiang S. (2021). Cordycepin ameliorates nonalcoholic steatohepatitis by activation of the AMP-activated protein kinase signaling pathway. Hepatology 74, 686–703. 10.1002/hep.31749 33576035 PMC8457150

[B29] Lee Y. S. Kim J. W. Osborne O. Oh D. Y. Sasik R. Schenk S. (2014). Increased adipocyte O2 consumption triggers HIF-1α, causing inflammation and insulin resistance in obesity. Cell 157, 1339–1352. 10.1016/j.cell.2014.05.012 24906151 PMC4114226

[B30] Li J. X. Li R. Z. Sun A. Zhou H. Neher E. Yang J. S. (2021). Metabolomics and integrated network pharmacology analysis reveal Tricin as the active anti-cancer component of Weijing decoction by suppression of PRKCA and sphingolipid signaling. Pharmacol. Res. 171, 105574. 10.1016/j.phrs.2021.105574 34419228

[B31] Li V. L. He Y. Contrepois K. Liu H. Kim J. T. Wiggenhorn A. L. (2022). An exercise-inducible metabolite that suppresses feeding and obesity. Nature 606, 785–790. 10.1038/s41586-022-04828-5 35705806 PMC9767481

[B32] Liu M. Huang Y. Zhang H. Aitken D. Nevitt M. C. Rockel J. S. (2022). Restricting branched-chain amino acids within a high-fat diet prevents obesity. Metabolites 12, 334. 10.3390/metabo12040334 35448521 PMC9030079

[B33] Liu Q. Hong I. P. Ahn M. J. Yoo H. S. Han S. B. Hwang B. Y. (2011). Anti-adipogenic activity of Cordyceps militaris in 3T3-L1 cells. Nat. Prod. Commun. 6, 1839–1841. 10.1177/1934578x1100601213 22312720

[B34] Liu T. Wang J. Tong Y. Wu L. Xie Y. He P. (2024). Integrating network pharmacology and animal experimental validation to investigate the action mechanism of oleanolic acid in obesity. J. Transl. Med. 22, 86. 10.1186/s12967-023-04840-x 38246999 PMC10802007

[B35] Li Y. Li Y. Wang X. Xu H. Wang C. An Y. (2018). Cordycepin modulates body weight by reducing prolactin via an adenosine A1 receptor. Curr. Pharm. Des. 24, 3240–3249. 10.2174/1381612824666180820144917 30124145

[B36] Li Y. Zou L. Li T. Lai D. Wu Y. Qin S. (2019). Mogroside V inhibits LPS-induced COX-2 expression/ROS production and overexpression of HO-1 by blocking phosphorylation of AKT1 in RAW264.7 cells. Acta Biochim. Biophys. Sin. (Shanghai) 51, 365–374. 10.1093/abbs/gmz014 30877761

[B37] Luo M. Mengos A. E. Stubblefield T. M. Mandarino L. J. (2012). High fat diet-induced changes in hepatic protein abundance in mice. J. Proteomics Bioinform 5, 60–66. 10.4172/jpb.1000214 33907358 PMC8074682

[B38] Lv S. Chen Q. Li Z. Zhou Z. (2021). An evidence update on the protective mechanism of tangeretin against neuroinflammation based on network pharmacology prediction and transcriptomic analysis. Eur. J. Pharmacol. 906, 174094. 10.1016/j.ejphar.2021.174094 34087222

[B39] Martinez-Huenchullan S. F. Shipsey I. Hatchwell L. Min D. Twigg S. M. Larance M. (2021). Blockade of high-fat diet proteomic phenotypes using exercise as prevention or treatment. Mol. Cell Proteomics 20, 100027. 10.1074/mcp.TIR120.002343 33594989 PMC7950115

[B40] Matesanz N. Nikolic I. Leiva M. PulgaríN-Alfaro M. Santamans A. M. Bernardo E. (2018). p38α blocks brown adipose tissue thermogenesis through p38δ inhibition. PLoS Biol. 16, e2004455. 10.1371/journal.pbio.2004455 29979672 PMC6051667

[B41] Meng D. Zhang B. Wang Y. Zheng T. Hu R. Wang B. (2022). p38α deficiency in T cells ameliorates diet-induced obesity, insulin resistance, and adipose tissue senescence. Diabetes 71, 1205–1217. 10.2337/db21-0653 35349644

[B42] Nakae J. Kitamura T. Kitamura Y. Biggs W. H. 3Rd Arden K. C. Accili D. (2003). The forkhead transcription factor Foxo1 regulates adipocyte differentiation. Dev. Cell 4, 119–129. 10.1016/s1534-5807(02)00401-x 12530968

[B43] Nesteruk M. Hennig E. E. Mikula M. Karczmarski J. Dzwonek A. Goryca K. (2014). Mitochondrial-related proteomic changes during obesity and fasting in mice are greater in the liver than skeletal muscles. Funct. Integr. Genomics 14, 245–259. 10.1007/s10142-013-0342-3 24178926 PMC3968515

[B44] Niu L. Geyer P. E. Wewer Albrechtsen N. J. Gluud L. L. Santos A. Doll S. (2019). Plasma proteome profiling discovers novel proteins associated with non-alcoholic fatty liver disease. Mol. Syst. Biol. 15, e8793. 10.15252/msb.20188793 30824564 PMC6396370

[B45] Oba D. Inoue S. I. Miyagawa-Tomita S. Nakashima Y. Niihori T. Yamaguchi S. (2018). Mice with an oncogenic HRAS mutation are resistant to high-fat diet-induced obesity and exhibit impaired hepatic energy homeostasis. EBioMedicine 27, 138–150. 10.1016/j.ebiom.2017.11.029 29254681 PMC5828294

[B46] Park J. C. Jeong W. J. Seo S. H. Choi K. Y. (2019). WDR76 mediates obesity and hepatic steatosis via HRas destabilization. Sci. Rep. 9, 19676. 10.1038/s41598-019-56211-6 31873167 PMC6927951

[B47] Peng S. Li W. Hou N. Huang N. (2020). A review of FoxO1-regulated metabolic diseases and related drug discoveries. Cells 9, 184. 10.3390/cells9010184 31936903 PMC7016779

[B49] Qi G. Zhou Y. Zhang X. Yu J. Li X. Cao X. (2019). Cordycepin promotes browning of white adipose tissue through an AMP-activated protein kinase (AMPK)-dependent pathway. Acta Pharm. Sin. B 9, 135–143. 10.1016/j.apsb.2018.10.004 30766785 PMC6361849

[B50] Rausch M. E. Weisberg S. Vardhana P. Tortoriello D. V. (2008). Obesity in C57BL/6J mice is characterized by adipose tissue hypoxia and cytotoxic T-cell infiltration. Int. J. Obes. (Lond) 32, 451–463. 10.1038/sj.ijo.0803744 17895881

[B51] Ross S. E. Erickson R. L. Hemati N. Macdougald O. A. (1999). Glycogen synthase kinase 3 is an insulin-regulated C/EBPalpha kinase. Mol. Cell Biol. 19, 8433–8441. 10.1128/MCB.19.12.8433 10567568 PMC84944

[B52] Shi L. Tao Z. Zheng L. Yang J. Hu X. Scott K. (2023). FoxO1 regulates adipose transdifferentiation and iron influx by mediating Tgfβ1 signaling pathway. Redox Biol. 63, 102727. 10.1016/j.redox.2023.102727 37156218 PMC10195981

[B53] Son J. W. Kim S. (2020). Comprehensive review of current and upcoming anti-obesity drugs. Diabetes and Metabolism J. 44, 802–818. 10.4093/dmj.2020.0258 PMC7801751 33389955

[B54] Song N. J. Chang S. H. Kim S. Panic V. Jang B. H. Yun U. J. (2018). PI3Ka-Akt1-mediated Prdm4 induction in adipose tissue increases energy expenditure, inhibits weight gain, and improves insulin resistance in diet-induced obese mice. Cell Death Dis. 9, 876. 10.1038/s41419-018-0904-3 30158592 PMC6115456

[B55] Takahashi S. Tamai M. Nakajima S. Kato H. Johno H. Nakamura T. (2012). Blockade of adipocyte differentiation by cordycepin. Br. J. Pharmacol. 167, 561–575. 10.1111/j.1476-5381.2012.02005.x 22537056 PMC3449261

[B56] Tan L. Song X. Ren Y. Wang M. Guo C. Guo D. (2020). Anti-inflammatory effects of cordycepin: a review. Phytother. Res. 35, 1284–1297. 10.1002/ptr.6890 33090621

[B57] Wan M. Easton R. M. Gleason C. E. Monks B. R. Ueki K. Kahn C. R. (2012). Loss of Akt1 in mice increases energy expenditure and protects against diet-induced obesity. Mol. Cell Biol. 32, 96–106. 10.1128/MCB.05806-11 22037765 PMC3255699

[B58] Wu W. D. Hu Z. M. Shang M. J. Zhao D. J. Zhang C. W. Hong D. F. (2014). Cordycepin down-regulates multiple drug resistant (MDR)/HIF-1α through regulating AMPK/mTORC1 signaling in GBC-SD gallbladder cancer cells. Int. J. Mol. Sci. 15, 12778–12790. 10.3390/ijms150712778 25046749 PMC4139874

[B59] Xiao S. Li V. L. Lyu X. Chen X. Wei W. Abbasi F. (2024). Lac-Phe mediates the effects of metformin on food intake and body weight. Nat. Metab. 6, 659–669. 10.1038/s42255-024-00999-9 38499766 PMC11062621

[B60] Xu H. Wu B. Wang X. Ma F. Li Y. An Y. (2019). Cordycepin regulates body weight by inhibiting lipid droplet formation, promoting lipolysis and recruiting beige adipocytes. J. Pharm. Pharmacol. 71, 1429–1439. 10.1111/jphp.13127 31259423

[B61] Yang X. Li Y. He Y. Li T. Wang W. Zhang J. (2015). Cordycepin alleviates airway hyperreactivity in a murine model of asthma by attenuating the inflammatory process. Int. Immunopharmacol. 26, 401–408. 10.1016/j.intimp.2015.04.017 25912153

[B62] Yen C. L. Chao W. C. Wu C. H. Huang Y. F. Chang C. S. Tsai Y. S. (2015). Phosphorylation of glycogen synthase kinase-3β in metabolically abnormal obesity affects immune stimulation-induced cytokine production. Int. J. Obes. (Lond) 39, 270–278. 10.1038/ijo.2014.93 24854430

[B63] Yoon S. Y. Park S. J. Park Y. J. (2018). The anticancer properties of cordycepin and their underlying mechanisms. Int. J. Mol. Sci. 19, 3027. 10.3390/ijms19103027 30287757 PMC6212910

[B64] Zhang H. Yang S. Wang J. Jiang Y. (2021). Blockade of AMPK-mediated cAMP-PKA-CREB/ATF1 signaling synergizes with aspirin to inhibit hepatocellular carcinoma. Cancers (Basel) 13, 1738. 10.3390/cancers13071738 33917483 PMC8038809

[B65] Zhang S. Cao H. Li Y. Jing Y. Liu S. Ye C. (2018). Metabolic benefits of inhibition of p38α in white adipose tissue in obesity. PLoS Biol. 16, e2004225. 10.1371/journal.pbio.2004225 29750781 PMC5965899

[B66] Zhang S. Cui Y. Gao X. Wei C. Wang Q. Yang B. (2023). Resveratrol inhibits the formation and accumulation of lipid droplets through AdipoQ signal pathway and lipid metabolism lncRNAs. J. Nutr. Biochem. 117, 109351. 10.1016/j.jnutbio.2023.109351 37087074

[B67] Zhang Y. Wu X. Xu M. Yue T. Ling P. Fang T. (2022). Comparative proteomic analysis of liver tissues and serum in db/db mice. Int. J. Mol. Sci. 23, 9687. 10.3390/ijms23179687 36077090 PMC9455973

[B68] Zhong L. P. Li J. Wang F. Z. Zhu H. B. Hou X. J. (2017). Protective effect and underlying mechanism of cordycepin on non-alcoholic fatty liver in ob/ob mice. Yao Xue Xue Bao 52, 106–112. 10.16438/j.0513-4870.2016-0886 29911800

