# Supplementary material for: Integrating network pharmacology, quantitative transcriptomic analysis, and experimental validation revealed the mechanism of cordycepin in the treatment of obesity
Source: Front Pharmacol. 2025 May 14;16:1571480. doi: 10.3389/fphar.2025.1571480 (PMC12116500; doi:10.3389/fphar.2025.1571480)
Supplement: Supplementary file 1 [file Supplementaryfile1.docx]

Original article

**Integrating network pharmacology, quantitative transcriptomic analysis, and experimental validation revealed the mechanism of cordycepin in the treatment of obesity**

**Supporting tables and figures**

**Table S1** 244 potential anti-obesity targets of cordycepin.

| Number | Target | Number | Target | Number | Target | Number | | Target |
| --- | --- | --- | --- | --- | --- | --- | --- | --- |
| 1 | ADORA2A | 66 | TERT | 131 | REG1A | 196 | FGG | |
| 2 | ADORA1 | 67 | TRPM2 | 132 | ARHGAP1 | 197 | BACE1 | |
| 3 | ADORA3 | 68 | FDFT1 | 133 | F2 | 198 | F11 | |
| 4 | DPP4 | 69 | PNP | 134 | AKR1B1 | 199 | ARF4 | |
| 5 | AHCY | 70 | ADH1A | 135 | SHMT1 | 200 | NT5M | |
| 6 | ADK | 71 | GGPS1 | 136 | PDE4D | 201 | WARS1 | |
| 7 | EHMT1 | 72 | EP300 | 137 | MMP3 | 202 | HADH | |
| 8 | ADORA2B | 73 | LDHB | 138 | SELP | 203 | AKT2 | |
| 9 | AMD1 | 74 | OGT | 139 | OTC | 204 | NR3C1 | |
| 10 | ADA | 75 | MMP9 | 140 | RAB11A | 205 | OAT | |
| 11 | SETD7 | 76 | CDC42 | 141 | B3GAT1 | 206 | KIF11 | |
| 12 | EHMT2 | 77 | RAC1 | 142 | PCK1 | 207 | SELE | |
| 13 | EGFR | 78 | PRKACA | 143 | CCL5 | 208 | PMS2 | |
| 14 | GAPDH | 79 | HSD17B1 | 144 | MMP12 | 209 | GP1BA | |
| 15 | SLC29A1 | 80 | P2RY2 | 145 | MTHFD1 | 210 | MAPK10 | |
| 16 | HSPA8 | 81 | PGD | 146 | CYP2C9 | 211 | KDR | |
| 17 | CA2 | 82 | POLB | 147 | SERPINA1 | 212 | GLTP | |
| 18 | CA1 | 83 | MAPK1 | 148 | BIRC7 | 213 | RAN | |
| 19 | CA12 | 84 | GRK1 | 149 | ERI1 | 214 | KAT2B | |
| 20 | CA9 | 85 | PRKAB1 | 150 | ELANE | 215 | GMPR2 | |
| 21 | SETD2 | 86 | PRKAG1 | 151 | FABP4 | 216 | GSTM2 | |
| 22 | CARM1 | 87 | ADCY5 | 152 | HRAS | 217 | GART | |
| 23 | PRMT1 | 88 | PRMT7 | 153 | PDE5A | 218 | HNMT | |
| 24 | HSPA5 | 89 | NR2F2 | 154 | PTPN1 | 219 | RAF1 | |
| 25 | PDCD4 | 90 | POLG | 155 | TPH1 | 220 | XIAP | |
| 26 | MCL1 | 91 | GPR17 | 156 | NMNAT1 | 221 | GCK | |
| 27 | FBP1 | 92 | MAP3K7 | 157 | LGALS3 | 222 | F10 | |
| 28 | DOT1L | 93 | PAX8 | 158 | ARG2 | 223 | GSTP1 | |
| 29 | SMS | 94 | PLCG1 | 159 | SULT1A1 | 224 | PCMT1 | |
| 30 | KMT2A | 95 | NPEPPS | 160 | PAPSS1 | 225 | MME | |
| 31 | DNMT1 | 96 | AHCYL1 | 161 | EPHA2 | 226 | GSTM1 | |
| 32 | EZH2 | 97 | TYMP | 162 | CHIT1 | 227 | TGFB2 | |
| 33 | SETDB1 | 98 | PDE4B | 163 | KIT | 228 | MAPK12 | |
| 34 | PNMT | 99 | CDK2 | 164 | MAPK14 | 229 | CLEC4M | |
| 35 | DNMT3B | 100 | PYGL | 165 | HCK | 230 | FKBP1B | |
| 36 | P2RY1 | 101 | AMY1A | 166 | CTSS | 231 | FOLH1 | |
| 37 | P2RY11 | 102 | AMY1B | 167 | IL2 | 232 | SULT2A1 | |
| 38 | IDO1 | 103 | AMY1C | 168 | INSR | 233 | TPSB2 | |
| 39 | NOS1 | 104 | BMP7 | 169 | DHFR | 234 | ACAT1 | |
| 40 | NOS2 | 105 | AZGP1 | 170 | ARG1 | 235 | CASP3 | |
| 41 | NOS3 | 106 | PIM1 | 171 | ZAP70 | 236 | DAPK1 | |
| 42 | ADAM17 | 107 | CTSB | 172 | MMP1 | 237 | ABL1 | |
| 43 | FHIT | 108 | PAH | 173 | ALDOA | 238 | HMGCR | |
| 44 | AR | 109 | RHOA | 174 | ABO | 239 | CASP1 | |
| 45 | SRC | 110 | MAN1B1 | 175 | RAB9A | 240 | RND3 | |
| 46 | HPRT1 | 111 | CTSK | 176 | CD209 | 241 | G6PD | |
| 47 | MAPKAPK2 | 112 | HK1 | 177 | CLK1 | 242 | BTK | |
| 48 | HSPA1A | 113 | CCNA2 | 178 | SULT1E1 | 243 | F7 | |
| 49 | AKT1 | 114 | ISG20 | 179 | GSTA1 | 244 | MAOB | |
| 50 | RNASEL | 115 | PDPK1 | 180 | APRT |  |  | |
| 51 | RNASE1 | 116 | PLAU | 181 | TPI1 |  |  | |
| 52 | P2RY12 | 117 | CSNK2A1 | 182 | REN |  |  | |
| 53 | ENPP1 | 118 | MMP8 | 183 | PTK2 |  |  | |
| 54 | ADCY10 | 119 | C1R | 184 | GSR |  |  | |
| 55 | TYMS | 120 | PDE3B | 185 | FGFR1 |  |  | |
| 56 | IMPDH2 | 121 | AMY2A | 186 | SULT2B1 |  |  | |
| 57 | EIF4E | 122 | LCK | 187 | AK1 |  |  | |
| 58 | DTYMK | 123 | RHEB | 188 | STAT1 |  |  | |
| 59 | HLCS | 124 | HSP90AA1 | 189 | GSTO1 |  |  | |
| 60 | BCHE | 125 | GSK3B | 190 | PKLR |  |  | |
| 61 | CD38 | 126 | RNASE4 | 191 | TK1 |  |  | |
| 62 | CHAT | 127 | PDHB | 192 | TRDMT1 |  |  | |
| 63 | CA14 | 128 | RAB5A | 193 | PLEKHA4 |  |  | |
| 64 | MMP2 | 129 | TREM1 | 194 | BCAT2 |  |  | |
| 65 | IMPDH1 | 130 | CHEK1 | 195 | NNT |  |  | |

**Table S2** Genes differentially expressed between the WD and Chow groups (p<0.05).

| Gene name | Log2 fold change | P-value | Gene name | Log2 fold change | P-value |
| --- | --- | --- | --- | --- | --- |
| Ampd1 | 4.118870741 | 0.01994 | Taldo1 | 0.479221713 | 0.016797 |
| Aldob | 3.426446817 | 0.015684 | Ndufs7 | 0.475685333 | 0.00544 |
| Pah | 2.918351487 | 0.012246 | Nup205 | 0.46716215 | 0.018823 |
| Bhmt2 | 2.806982583 | 0.008226 | Gns | 0.454623394 | 0.004991 |
| Apoa2 | 2.764231559 | 0.017773 | Rragc | 0.448648069 | 0.028404 |
| Apoa1 | 2.621460284 | 0.022357 | Atp5d | 0.417761493 | 0.034249 |
| Fdx1 | 2.613249151 | 0.017005 | H2 | 0.417518053 | 0.008448 |
| Cps1 | 2.569433966 | 0.008075 | Uqcrq | 0.40052705 | 0.027748 |
| Thbs2 | 2.556443752 | 0.010446 | Psmc1 | 0.399256752 | 0.003166 |
| Apoc3 | 2.522088355 | 0.028275 | Ndufa3 | 0.384892616 | 0.031559 |
| Thbs1 | 2.237033525 | 0.019349 | Sqstm1 | 0.351116471 | 0.000112 |
| Bhmt2 | 2.232905334 | 0.009391 | Herc1 | 0.331738861 | 0.021003 |
| Cyp4a10 | 2.186356503 | 0.005593 | H2 | 0.330848775 | 0.031711 |
| Ak1 | 2.161617101 | 0.006036 | Stat6 | 0.317831979 | 0.018885 |
| Apom | 2.121921721 | 0.030811 | Ppm1a | 0.306649688 | 0.012685 |
| Haao | 2.055790378 | 0.032362 | Chmp6 | 0.296471664 | 0.037616 |
| Mat1a | 1.935826138 | 0.039126 | Psmb1 | 0.285447323 | 0.036262 |
| Ttr | 1.872214557 | 0.02234 | Pgd | 0.27776336 | 0.039088 |
| Pdk4 | 1.852695501 | 0.035879 | Cops6 | 0.276218238 | 0.003494 |
| Sds | 1.656255501 | 0.032969 | Map1lc3b | 0.26263717 | 0.027578 |
| Nox1 | 1.602866064 | 0.000876 | Ndufa2 | 0.255419499 | 0.003275 |
| H2 | 1.601105329 | 0.024282 | Sod1 | 0.246682671 | 0.008003 |
| Ms4a4a | 1.542920286 | 0.018014 | Selenok | 0.207642371 | 0.027495 |
| Hpd | 1.538555945 | 0.041764 | Psma3 | 0.155361275 | 0.044019 |
| Apoc2 | 1.517669437 | 0.036663 | Hsf1 | 0.139945584 | 0.048025 |
| Fah | 1.459814258 | 0.047561 | Gapvd1 | -0.163705925 | 0.004045 |
| Aldoa | 1.431665058 | 0.001148 | Ash1l | -0.184973073 | 0.016559 |
| Hmox1 | 1.415924815 | 0.017124 | Stam2 | -0.241126758 | 0.023476 |
| Nox4 | 1.396430819 | 0.02215 | Rps6kb2 | -0.267616694 | 0.029346 |
| Prodh2 | 1.36960143 | 0.006026 | Arf5 | -0.294792436 | 0.043523 |
| Idh3a | 1.352413096 | 0.022554 | Mapk8 | -0.301345606 | 0.038862 |
| Cd163 | 1.295876944 | 0.042415 | Brcc3 | -0.3116996 | 0.012003 |
| Apoe | 1.233069755 | 0.041345 | Akt3 | -0.338333342 | 0.042178 |
| Acaa2 | 1.200076509 | 0.007973 | Rest | -0.356342082 | 0.046471 |
| Egfr | 1.180950824 | 0.029005 | Mlst8 | -0.427364315 | 0.042292 |
| Ttpa | 1.17303742 | 0.034407 | Ar | -0.441084883 | 0.024143 |
| Gapdh | 1.116478228 | 0.002941 | Pik3r3 | -0.447197566 | 0.008009 |
| H6pd | 1.101187361 | 0.046365 | Nfat5 | -0.453612276 | 0.007514 |
| Gpx1 | 1.063805422 | 0.017213 | Arid1a | -0.457516373 | 0.000304 |
| Mgst3 | 1.061097776 | 0.034105 | Pik3c2a | -0.499980185 | 0.026616 |
| Cox8a | 1.057056265 | 0.021656 | Ccl5 | -0.515292293 | 0.010816 |
| Sdsl | 1.012362703 | 0.01518 | Arpc4 | -0.534522947 | 0.018036 |
| Acadl | 1.006847424 | 0.004279 | Jak2 | -0.595441103 | 0.017186 |
| Glyctk | 0.935527574 | 0.020387 | Rpia | -0.603977044 | 0.040332 |
| Ctsz | 0.91286089 | 0.031942 | Braf | -0.607745467 | 0.000334 |
| S100a1 | 0.900801014 | 0.046034 | Ppat | -0.623239954 | 0.034867 |
| Icos | 0.886352769 | 0.029856 | Dera | -0.64552639 | 0.049259 |
| Ldha | 0.885248186 | 0.017859 | Itk | -0.663434303 | 0.031005 |
| Adcy2 | 0.877871793 | 0.045216 | Cd244a | -0.698841964 | 0.038518 |
| Hadh | 0.86766707 | 0.024358 | Srebf2 | -0.705894764 | 0.023961 |
| Flt1 | 0.859017552 | 0.045782 | Acy1 | -0.708229213 | 0.035143 |
| Sdhb | 0.857570021 | 0.000629 | Mapk8ip1 | -0.712072855 | 0.044162 |
| Gpx4 | 0.847836443 | 0.028823 | Cox7b | -0.775734881 | 0.027203 |
| Acat1 | 0.831612813 | 0.042871 | Gclc | -0.817664036 | 0.029397 |
| Ctss | 0.80403047 | 0.030165 | Nos2 | -0.849204172 | 0.022635 |
| H2 | 0.800935495 | 0.038128 | Ctsw | -0.874451444 | 0.035532 |
| Ogdh | 0.780739795 | 0.01118 | Gtse1 | -0.894109766 | 0.000631 |
| Sdhc | 0.759737405 | 0.001769 | Aoc1 | -0.923657699 | 0.000303 |
| Sardh | 0.747714334 | 0.017265 | Lck | -0.960295605 | 0.019567 |
| Npr1 | 0.714213496 | 0.049679 | Zfp458 | -0.971257489 | 0.026887 |
| Echs1 | 0.706575306 | 0.037023 | Slc16a8 | -1.021074594 | 0.048163 |
| Fh1 | 0.696240451 | 0.004415 | Prim1 | -1.061011679 | 0.001563 |
| Idh3g | 0.692445046 | 0.002346 | Cd3g | -1.070634141 | 0.000513 |
| Bcl2l1 | 0.66386003 | 0.044236 | Slc16a2 | -1.089000918 | 0.039048 |
| Fabp5 | 0.659638663 | 0.046715 | Tyms | -1.123336188 | 0.003499 |
| Tlr4 | 0.656081433 | 0.033605 | Cd3e | -1.123854158 | 0.001039 |
| Cs | 0.645250505 | 0.002378 | Ido2 | -1.138688432 | 0.015254 |
| Uqcr10 | 0.634062179 | 0.023415 | Neu1 | -1.183969512 | 0.018779 |
| Cox5a | 0.624140506 | 0.022237 | Il2 | -1.282646754 | 0.012991 |
| Umps | 0.620771528 | 0.031286 | Flt3 | -1.308365392 | 0.004385 |
| Cox7c | 0.615365901 | 0.003254 | Tph1 | -1.323093765 | 6.01E-05 |
| Ampd3 | 0.609870124 | 0.043792 | Tnf | -1.406601299 | 0.003892 |
| Deptor | 0.606873708 | 0.0278 | Rrm2 | -1.473357662 | 9.92E-05 |
| Acsf3 | 0.595313851 | 0.007356 | Cd8b1 | -1.487528394 | 0.001252 |
| Itgb5 | 0.587425927 | 0.022461 | Fanci | -1.509418325 | 0.014973 |
| Idh3b | 0.578826958 | 0.024506 | Krt1 | -1.512853257 | 0.009389 |
| Pten | 0.56744998 | 0.042947 | Got2 | -1.578097733 | 0.039169 |
| Atg101 | 0.560352935 | 0.026635 | Aanat | -1.630120713 | 0.008077 |
| Cox6a1 | 0.551179384 | 0.046507 | Ccnd1 | -1.726700174 | 0.01857 |
| Nadk | 0.5461424 | 0.027131 | Cdca5 | -1.767050564 | 0.011113 |
| Ndufb8 | 0.539104796 | 0.00791 | Efna4 | -1.82343015 | 0.017331 |
| Irf1 | 0.537774601 | 0.028624 | Rad51 | -1.858324408 | 0.009622 |
| Ak3 | 0.537704749 | 0.018113 | Ern1 | -1.8948631 | 0.03964 |
| Ndufb4 | 0.517095894 | 0.016948 | Shmt2 | -2.002550443 | 0.015132 |
| Cox5b | 0.515281732 | 0.04104 | Traf1 | -2.166587716 | 0.028134 |
| Uqcr11 | 0.512973891 | 0.027506 | Trp63 | -2.173974154 | 0.000506 |
| Ndufa4 | 0.512579856 | 0.043754 | H2 | -2.367998536 | 5.53E-05 |
| Pkm | 0.508873232 | 0.0413 | Slc7a11 | -2.405565448 | 0.017755 |
| Ndufa13 | 0.503258973 | 0.001925 | Alox12 | -2.771294899 | 0.025577 |
| Mlycd | 0.500080977 | 0.000154 | Folr1 | -2.823701721 | 0.034009 |
| Serinc1 | 0.49119703 | 0.024632 | Psph | -3.034082556 | 0.005654 |
| Pgk1 | 0.487373887 | 0.014531 | Fanca | -3.634686618 | 0.000436 |
| Nfs1 | 0.48613802 | 0.011539 | Pycr1 | -3.851768363 | 0.023743 |
| Cd63 | 0.484977176 | 0.025744 |  |  |  |

**Table S3** Genes differentially expressed between Cpn and WD groups (p<0.05).

| Gene name | Log2 fold change | P-value | Gene name | Log2 fold change | P-value |
| --- | --- | --- | --- | --- | --- |
| Trp63 | 1.956406879 | 0.006961 | Bcl2l1 | -0.555891075 | 0.003538 |
| Lamtor4 | 1.481450509 | 0.013817 | Asl | -0.653395707 | 0.028855 |
| Xcl1 | 0.899877075 | 0.044126 | Glyctk | -0.763833953 | 0.027133 |
| Cd8a | 0.859989829 | 0.03819 | Mycl | -0.860233682 | 0.035225 |
| Tph1 | 0.664131048 | 0.03016 | Acmsd | -1.081113175 | 0.049764 |
| Wnt1 | 0.65115997 | 0.024969 | Irf4 | -1.568449539 | 0.008376 |
| Srebf1 | 0.648703579 | 0.046884 | Tdo2 | -1.620764121 | 0.032406 |
| Ctsa | 0.601996751 | 0.021654 | Mat1a | -1.796007931 | 0.044629 |
| Pik3r1 | 0.57279547 | 0.013298 | Haao | -1.81275913 | 0.046559 |
| Rad51 | 0.564390769 | 0.002214 | Bhmt2 | -1.868646497 | 0.038167 |
| Cox7b | 0.46382616 | 0.039427 | Cyp8b1 | -2.00732451 | 0.008993 |
| Ppat | 0.445083189 | 0.005947 | Cps1 | -2.056191082 | 0.027872 |
| Hjurp | 0.422654595 | 0.043372 | Apob | -2.146372783 | 0.01749 |
| Cenpa | 0.420257036 | 0.011903 | Apom | -2.208513194 | 0.022499 |
| Msh2 | 0.304354934 | 0.025785 | Apoc3 | -2.253966121 | 0.042958 |
| Cab39 | 0.280329496 | 0.042495 | Apoa1 | -2.26881089 | 0.033454 |
| Prkag1 | 0.265809211 | 0.03146 | Cyp4a10 | -2.282450488 | 0.002155 |
| Brcc3 | 0.214402981 | 0.038061 | G6pc | -2.323461966 | 0.038366 |
| Selenok | -0.10310835 | 0.034714 | Cyp4a12a | -2.422984798 | 0.018903 |
| Myc | -0.43170226 | 0.034753 | Pah | -2.561860167 | 0.023471 |
| Flt1 | -0.487207772 | 0.033912 | Apoa2 | -2.655177168 | 0.025827 |
| Atg101 | -0.494786358 | 0.039914 | Rgn | -2.71588184 | 0.049733 |
| Slc3a2 | -0.548566648 | 0.009157 | Aldob | -3.372461944 | 0.016989 |
| Gpx1 | -0.55540085 | 0.025612 |  |  |  |

**Table S4** KEGG pathway analysis of differential genes in Cpn and WD groups

| Pathway ID | Pathway | out (42) | All (9181) | P-value |
| --- | --- | --- | --- | --- |
| ko03320 | PPAR signaling pathway | 6 | 92 | 3.37E-06 |
| ko01230 | Biosynthesis of amino acids | 5 | 80 | 2.93E-05 |
| ko01100 | Metabolic pathways | 19 | 1635 | 3.57E-05 |
| ko04979 | Cholesterol metabolism | 4 | 50 | 7.48E-05 |
| ko00380 | Tryptophan metabolism | 4 | 52 | 8.74E-05 |
| ko04152 | AMPK signaling pathway | 5 | 126 | 0.00025465 |
| ko00030 | Pentose phosphate pathway | 3 | 33 | 0.0004415 |
| ko00250 | Alanine, aspartate and glutamate metabolism | 3 | 38 | 0.00067187 |
| ko04150 | mTOR signaling pathway | 5 | 160 | 0.00076304 |
| ko04931 | Insulin resistance | 4 | 112 | 0.00164377 |
| ko01200 | Carbon metabolism | 4 | 121 | 0.00218267 |
| ko04391 | Hippo signaling pathway - fly | 3 | 64 | 0.00305558 |
| ko04910 | Insulin signaling pathway | 4 | 139 | 0.00360573 |
| ko00220 | Arginine biosynthesis | 2 | 20 | 0.00368439 |
| ko05212 | Pancreatic cancer | 3 | 76 | 0.00496365 |
| ko05220 | Chronic myeloid leukemia | 3 | 76 | 0.00496365 |
| ko04977 | Vitamin digestion and absorption | 2 | 25 | 0.00573392 |
| ko04932 | Non-alcoholic fatty liver disease | 4 | 159 | 0.00581223 |
| ko01524 | Platinum drug resistance | 3 | 82 | 0.0061355 |
| ko00790 | Folate biosynthesis | 2 | 29 | 0.00767075 |
| ko00590 | Arachidonic acid metabolism | 3 | 89 | 0.00769586 |
| ko05210 | Colorectal cancer | 3 | 90 | 0.00793616 |
| ko05225 | Hepatocellular carcinoma | 4 | 175 | 0.00812074 |
| ko04211 | Longevity regulating pathway | 3 | 91 | 0.00818087 |
| ko05222 | Small cell lung cancer | 3 | 96 | 0.00947113 |
| ko04066 | HIF-1 signaling pathway | 3 | 117 | 0.01614073 |
| ko04975 | Fat digestion and absorption | 2 | 44 | 0.01711732 |
| ko05417 | Lipid and atherosclerosis | 4 | 221 | 0.01789238 |
| ko03440 | Homologous recombination | 2 | 46 | 0.0186204 |
| ko04973 | Carbohydrate digestion and absorption | 2 | 50 | 0.021787 |
| ko04750 | Inflammatory mediator regulation of TRP channels | 3 | 132 | 0.02217833 |
| ko04068 | FoxO signaling pathway | 3 | 134 | 0.02306475 |
| ko00071 | Fatty acid degradation | 2 | 53 | 0.02429819 |
| ko00270 | Cysteine and methionine metabolism | 2 | 55 | 0.02603527 |
| ko04550 | Signaling pathways regulating pluripotency of stem cells | 3 | 141 | 0.02631848 |
| ko04140 | Autophagy - animal | 3 | 147 | 0.02929449 |
| ko05224 | Breast cancer | 3 | 149 | 0.03032479 |
| ko05213 | Endometrial cancer | 2 | 60 | 0.03059093 |
| ko05226 | Gastric cancer | 3 | 150 | 0.03084711 |
| ko04213 | Longevity regulating pathway - multiple species | 2 | 63 | 0.03346544 |
| ko00400 | Phenylalanine, tyrosine and tryptophan biosynthesis | 1 | 8 | 0.0360302 |
| ko05200 | Pathways in cancer | 6 | 549 | 0.0372999 |
| ko05206 | MicroRNAs in cancer | 3 | 162 | 0.03748576 |
| ko00010 | Glycolysis / Gluconeogenesis | 2 | 69 | 0.03951686 |
| ko04920 | Adipocytokine signaling pathway | 2 | 71 | 0.04162018 |
| ko05230 | Central carbon metabolism in cancer | 2 | 71 | 0.04162018 |
| ko05221 | Acute myeloid leukemia | 2 | 73 | 0.04376506 |
| ko04630 | JAK-STAT signaling pathway | 3 | 179 | 0.04804619 |

**Table S5** Molecular docking results of cordycepin with target proteins.

| Drug | Targets | Uniprot ID | PDB/Alphafold ID | Docking·score (kcal/mol) |
| --- | --- | --- | --- | --- |
| Cordycepin | CPS1 | Q8C196 | AF-Q8C196-F1-model_v4 | -8.6 |
|  | HRAS | Q61411 | 6kyh | -8.2 |
|  | GAPDH | P16858 | 6lgj | -7.6 |
|  | MAPK14 | P47811 | 1yw2 | -7.4 |
|  | PAH | P16331 | AF-P16331-F1-model_v4 | -7.4 |
|  | MMP9 | P41245 | AF-P41245-F1-model_v4 | -7.2 |
|  | SRC | P05480 | 6f3f | -7.2 |
|  | ALDOB | Q3UER1 | AF-Q3UER1-F1-model_v4 | -7.0 |
|  | AKT1 | P31750 | AF-P31750-F1-model_v4 | -7.0 |
|  | GSK3B | Q9WV60 | 6ae3 | -6.9 |
|  | HSP90AA1 | P07901 | 5h22 | -6.8 |
|  | BHMT2 | Q91WS4 | AF-Q91WS4-F1-model_v4 | -6.7 |
|  | EGFR | Q01279 | AF-Q01279-F1-model_v4 | -6.5 |
|  | CASP3 | P70677 | AF-P70677-F1-model_v4 | -6.4 |
|  | MAT1A | Q91X83 | AF-Q91X83-F1-model_v4 | -6.2 |
|  | APOM | Q9Z1R3 | 2xkl | -5.7 |
|  | APOA2 | P09813 | AF-P09813-F1-model_v4 | -5.7 |
|  | APOC3 | P33622 | AF-P33622-F1-model_v4 | -5.3 |
|  | APOA1 | Q00623 | AF-Q00623-F1-model_v4 | -4.9 |

**Table S6** Molecular docking results of cordycepin 5’-monophosphate with target proteins.

| Drug | Targets | Uniprot ID | PDB/Alphafold ID | Docking·score (kcal/mol) |
| --- | --- | --- | --- | --- |
|  | PAH | P16331 | AF-P16331-F1-model_v4 | -8.6 |
| Cordycepin 5’-monophosphate | MMP9 | P41245 | AF-P41245-F1-model_v4 | -8.4 |
|  | CPS1 | Q8C196 | AF-Q8C196-F1-model_v4 | -8.3 |
|  | AKT1 | P31750 | AF-P31750-F1-model_v4 | -8.2 |
|  | GSK3B | Q9WV60 | 6ae3 | -8.2 |
|  | HRAS | Q61411 | 6kyh | -8.1 |
|  | ALDOB | Q3UER1 | AF-Q3UER1-F1-model_v4 | -8.0 |
|  | SRC | P05480 | 6f3f | -7.8 |
|  | MAPK14 | P47811 | 1yw2 | -7.5 |
|  | GAPDH | P16858 | 6lgj | -7.4 |
|  | CASP3 | P70677 | AF-P70677-F1-model_v4 | -7.3 |
|  | HSP90AA1 | P07901 | 5h22 | -7.1 |
|  | EGFR | Q01279 | AF-Q01279-F1-model_v4 | -7.0 |
|  | BHMT2 | Q91WS4 | AF-Q91WS4-F1-model_v4 | -6.9 |
|  | MAT1A | Q91X83 | AF-Q91X83-F1-model_v4 | -6.8 |
|  | APOM | Q9Z1R3 | 2xkl | -6.3 |
|  | APOC3 | P33622 | AF-P33622-F1-model_v4 | -5.8 |
|  | APOA1 | Q00623 | AF-Q00623-F1-model_v4 | -5.3 |
|  | APOA2 | P09813 | AF-P09813-F1-model_v4 | -5.2 |


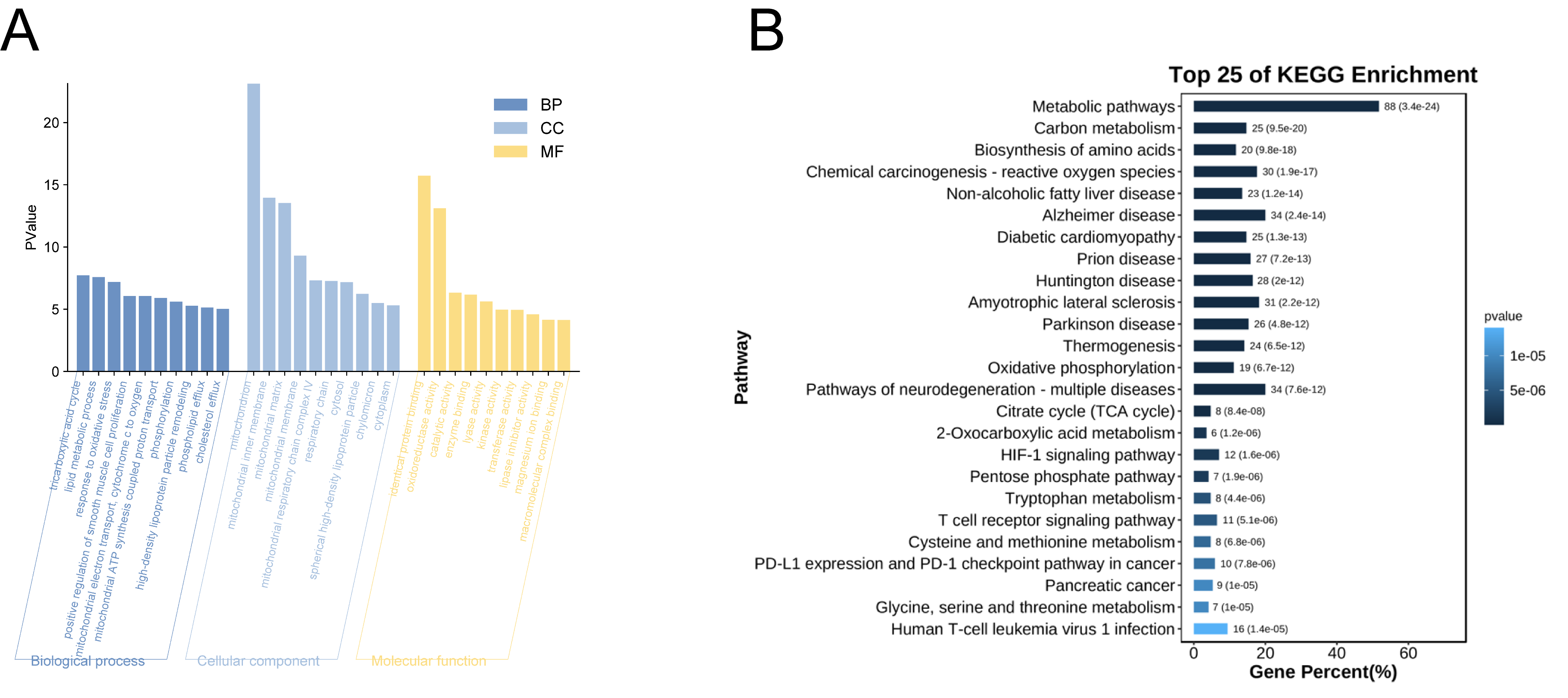


**Fig. S1** GO enrichment analysis and KEGG pathway analysis of differential genes in the Western diet (WD) and chow diet (Chow) groups (A) GO enrichment analysis. (B) KEGG pathway analysis.


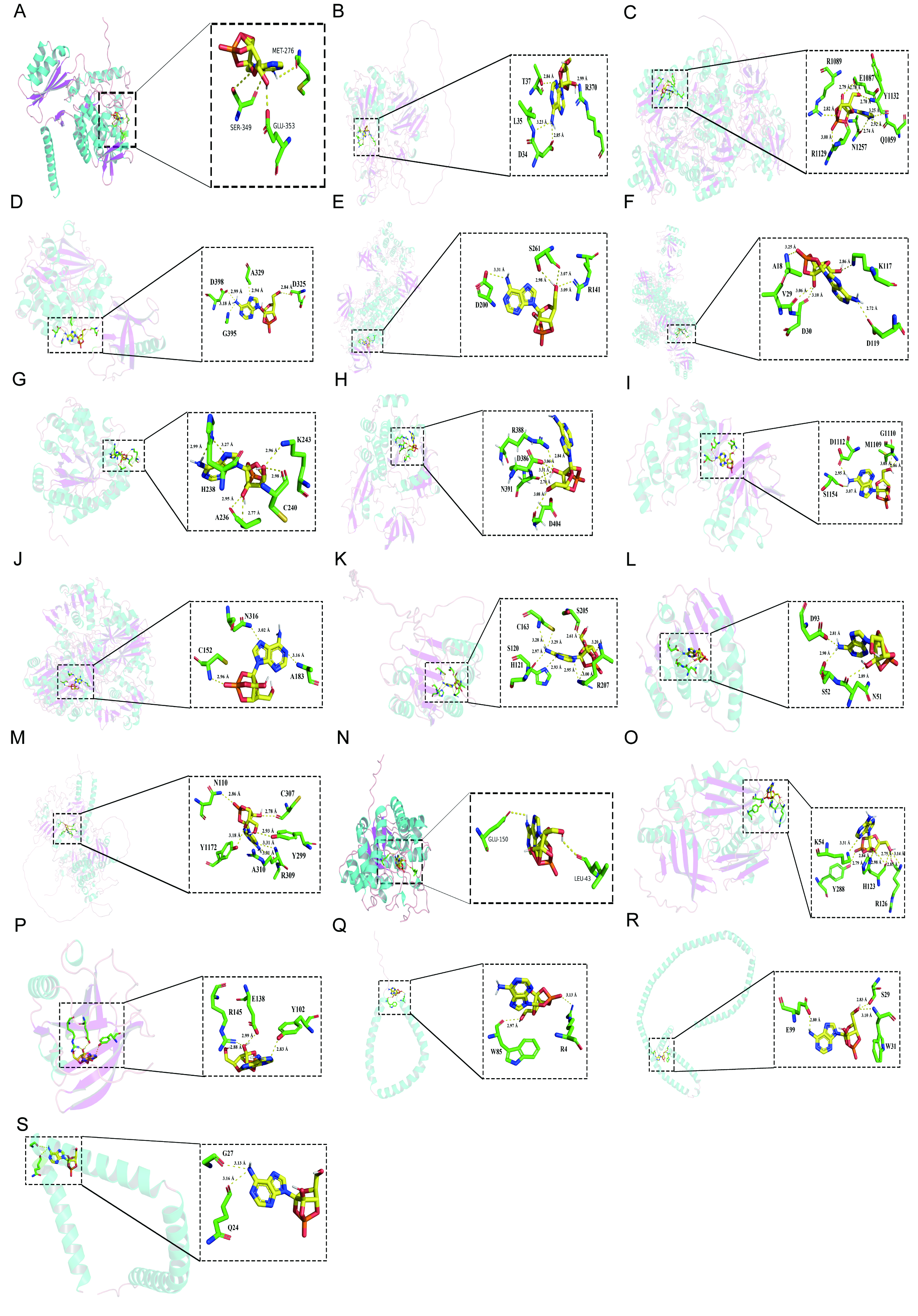


Fig. S2 The 3-dimensional map of the binding sites between cordycepin monophosphate and target proteins. (A) PAH. (B) MMP9. (C) CPS1. (D) AKT1. (E) GSK3B. (F) HRAS. (G) ALDOB. (H) SRC. (I) MAPK14. (J) GAPDH. (K) CASP3. (L) HSP90AA1. (M) EGFR. (N) BHMT2. (O) MAT1A. (P) APOM. (Q) APOC3. (R) APOA1. (S) APOA2.
