# Supplementary figures and images for: Integrating network pharmacology, quantitative transcriptomic analysis, and experimental validation revealed the mechanism of cordycepin in the treatment of obesity
Source: Front Pharmacol. 2025 May 14;16:1571480. doi: 10.3389/fphar.2025.1571480 (PMC12116500; doi:10.3389/fphar.2025.1571480)

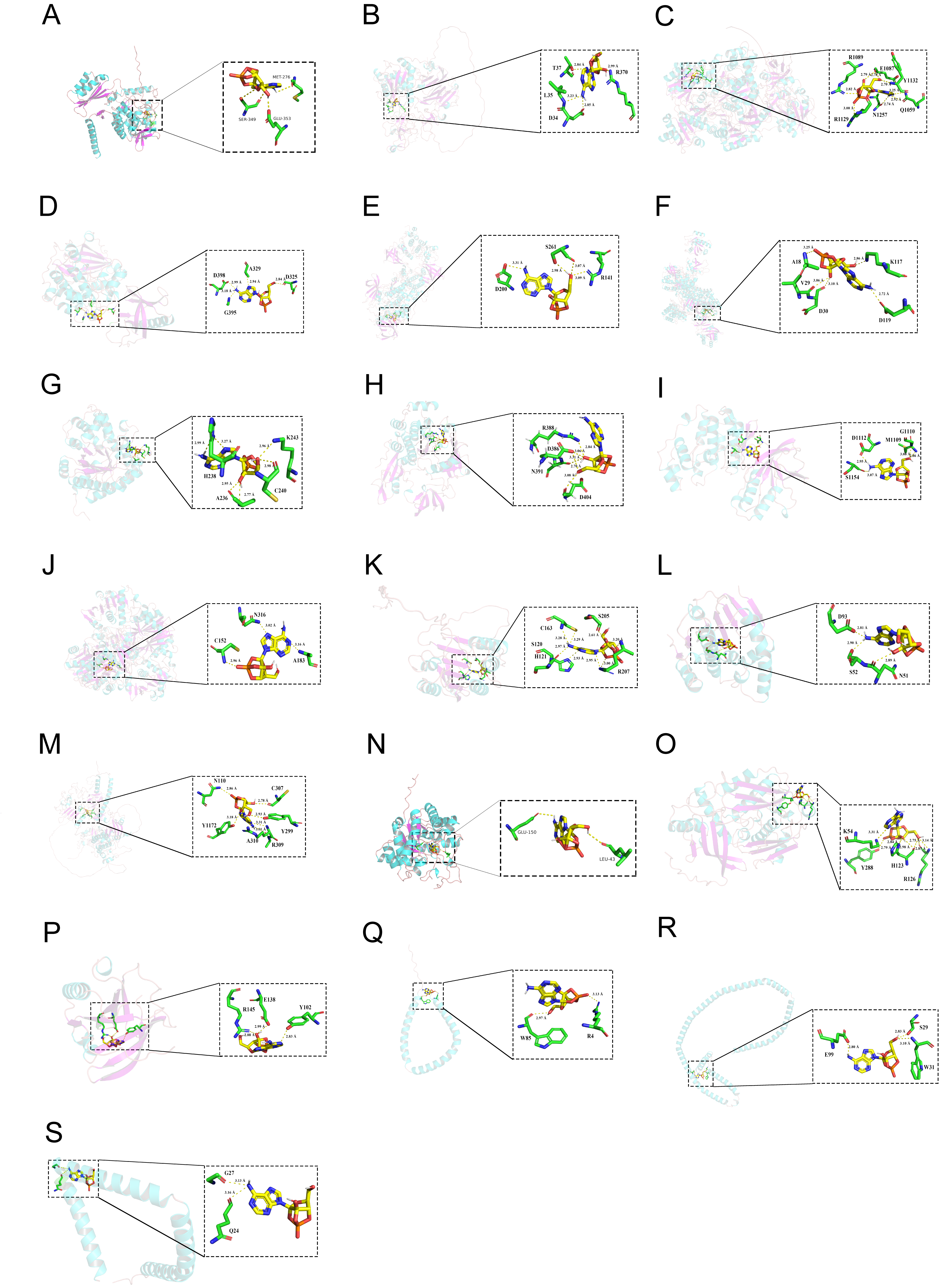

Supplement: Supplementary file 2 [file Image2.tif]

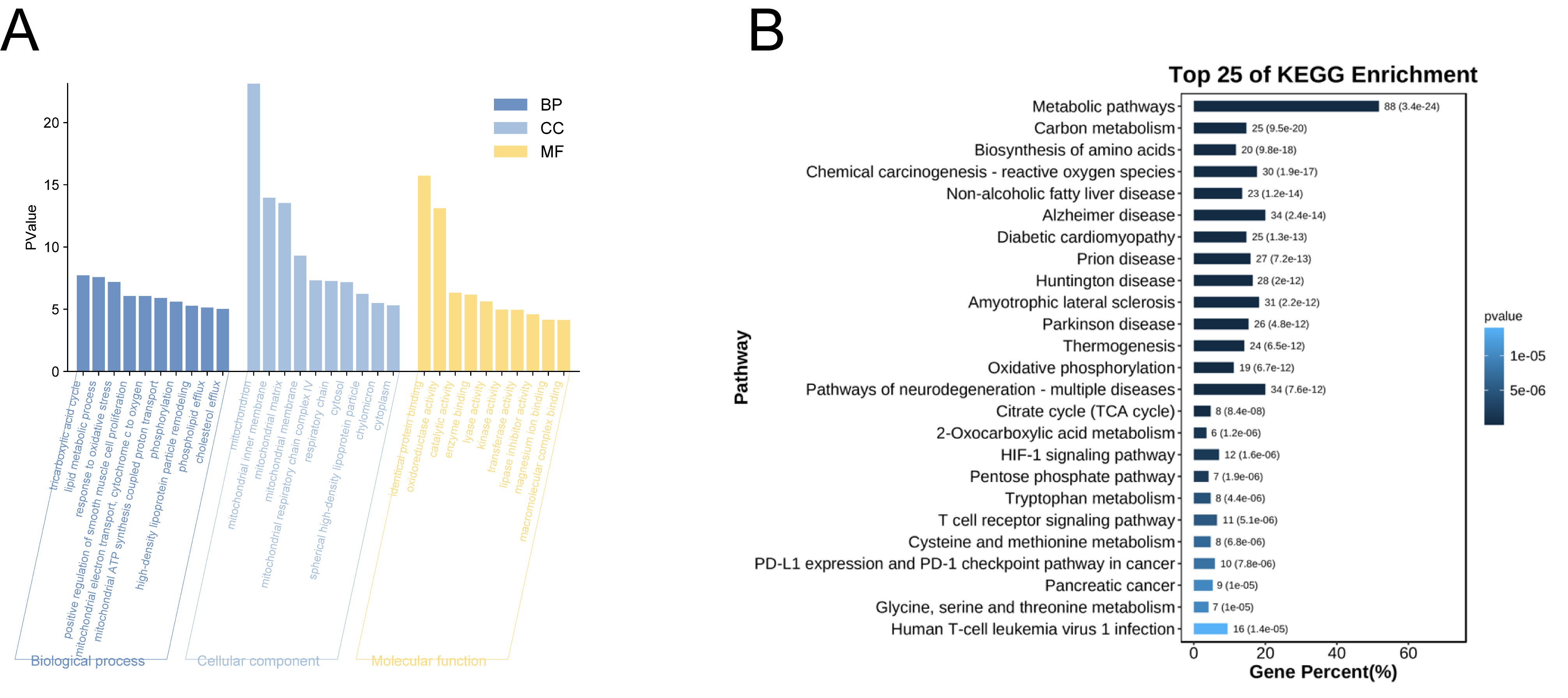

Supplement: Supplementary file 3 [file Image1.tif]
